# Clinical and laboratory manifestations, ECG findings, and outcomes of right atrial myxoma: a systematic review of cases reported worldwide

**DOI:** 10.1186/s43044-024-00550-x

**Published:** 2024-09-12

**Authors:** Mehrdad Rabiee Rad, Ghazal Ghasempour Dabaghi, Bahar Darouei, Reza Amani-Beni, Mohammad Mehdi Zare, Fatemeh Shirin, Marjan Jamalian

**Affiliations:** 1 Isfahan Cardiovascular Research Center, Cardiovascular Research Institute, Isfahan University of Medical Sciences, Isfahan, Iran; 2 School of Medicine, Isfahan University of Medical Science, Isfahan, Iran

**Keywords:** Right atrial myxoma, Atrial myxoma, Cardiac myxoma, Cardiac tumor, Systematic review

## Abstract

**Background:**

The presence of cardiac myxoma in the right atrium (RA) is rare. There is limited knowledge regarding the clinical symptoms and diagnosis of RA myxoma. This systematic review aimed to provide a summary of the clinical and laboratory characteristics, electrocardiogram (ECG) findings, and outcome previous cases with RA myxoma.

**Methods:**

A comprehensive search was conducted in PubMed, Web of Science, and Scopus to identify relevant studies. Inclusion criteria were case reports and case series written in English that provided sufficient data on the manifestation of RA myxoma. Descriptive statistics were used for quantitative analysis.

**Results:**

The search identified 619 patients from 480 eligible studies. The patient’s mean age was 45.7 ± 17.6 years, and 55.4% of cases were female. The most common clinical manifestations of RA myxoma were cardiac, systemic, and neurologic manifestations which reported in 77.0%, 34.8%, and 21.1% of cases, respectively. Besides, 11.7% of RA myxoma were asymptomatic. ECG findings revealed normal in 39.4% reported cases. The ECG abnormalities included tall or peaked P-wave, RA and LA enlargement (19.2%), abnormal T-wave (14.0%), sinus tachycardia (11.8%), and incomplete or complete RBBB (11.2%). Echocardiography remained the diagnostic method in a majority of the cases. The mortality rate of RA myxoma was low (9.2%) during the follow-up.

**Conclusions:**

This systematic review provides a comprehensive summary of the clinical and laboratory manifestations and outcomes of RA myxoma, contributing to the existing knowledge on this rare cardiac tumor.

**Supplementary Information:**

The online version contains supplementary material available at 10.1186/s43044-024-00550-x.

## Background

Cardiac masses are a rare group of disorders, with primary tumors and metastatic cancers being the main types. Primary cardiac masses account for about 1 out of 2000 autopsies and consist of both benign (80%) and malignant (20%) tumors [1, 2]. Among benign cardiac neoplasms, myxoma is the most common type, with a prevalence of 50% [3].

The symptoms of cardiac myxoma can vary depending on factors such as location, size, shape, and growth rate [4]. Patients with cardiac myxoma may experience multiple embolic events, such as stroke and pulmonary thromboembolism, due to the immobility of the tumor leading to infarctions [4, 5]. In addition, neurologic symptoms like syncope, psychiatric presentations, headache, and seizures are also common in patients with cardiac myxoma [6]. Cutaneous manifestations of cardiac myxoma can included erythematous, livedo reticularis, and macules [7, 8]. Hence, these varied symptoms and manifestations can make the diagnosis of cardiac myxoma challenging, as they can often mimic other conditions such as heart failure, arrhythmias, or even psychiatric disorders. Therefore, obtaining a thorough medical history, conducting a physical examination, and performing necessary diagnostic tests such as echocardiography, cardiac MRI, or biopsy are crucial for an accurate diagnosis of cardiac myxoma and to determine the optimal treatment approach.

Although there is previous research investigating left atrial myxoma, mitral valve, and pulmonary valve myxomas [9–11], prior attention to right atrial (RA) myxoma is limited. A study by Kuon et al. [12] suggests that RA myxoma can have various symptoms like lasting fever, weight loss, chronic anemia, and general arthralgia. Infection of myxoma and myocardial tamponade can develop because of misdiagnosing several complications of this disease like pulmonary hypertension, a Budd-Chiari syndrome with acute abdominal pain. These complications caused by misdiagnosis underline the need for a detailed evaluation of the right RA myxoma to prevent misdiagnoses and complications. In this systematic review, we aimed to summarize demographic data, signs and symptoms, laboratory and electrocardiogram (ECG) findings, and diagnostic methods published in previous case reports to claim better insight into this rare condition.

## Methods

This study was conducted following the Preferred Reporting Items for Systematic Reviews and Meta-Analysis (PRISMA) guideline [13].

### Search strategy

To identify eligible studies, a comprehensive search was conducted in September 2022 on PubMed, Web of Science, and Scopus. The search used the keywords “Cardiac myxoma” OR “Atrial myxoma” OR “Right atrial myxoma.” Additionally, a thorough reviews of the reference lists of qualified articles and google scholar were performed to identify any relevant missing articles.

### Eligible criteria

The screening process of the articles involved two reviewers assessing the titles, abstracts, and full-texts, with conflicts determined by another reviewer. Inclusion criteria encompassed case reports and case series written in English that provided sufficient data on the manifestation of right atrial myxoma. Exclusion criteria included books, editorials, reviews, experimental studies, congress abstracts, non-English articles, and case reports and case series that lacked adequate information on the clinical manifestation of right atrial myxoma. Studies involving animals were also excluded. There were no limitations on publication time or the age of participants.

### Data extraction

Data extraction was performed separately by two reviewers, incorporating the following categories: (i) bibliographic information (author, year of publication, study type, and country), (ii) demographics (number of patients, gender, age, and comorbidities), (iii) clinical manifestations categorized as cardiac, thromboembolic, neurologic, cutaneous, and systemic, (iv) diagnostic methods, (v) laboratory data (white blood cell (WBC), international normalized ratio (INR), erythrocyte sedimentation rate (ESR), C-reactive protein (CRP), troponin, and aminoterminal pro B-type natriuretic peptide (NT-proBNP)), (vi) ECG findings, and (vii) outcome. An elevated WBC count is defined as a count greater than 11,000 cells/μL [14]. The normal range for ESR in women is 0–20 mm/h, while in men it is 0–15 mm/h [15]. A CRP level higher than 0.9 mg/dL is considered elevated [16]. The normal limit for troponin-I is considered to be under 0.04 ng/Ml and troponin-T is considered to be under 0.01 ng/Ml [17]. Any conflicts or disagreements during the data extraction process were addressed and resolved by another reviewer. The clinical manifestations are divided into five groups including cardiac (palpitation, dyspnea, chest pain, raised jugular venous pressure, and ankle edema and hepatomegaly due to right-sided heart failure), thromboembolic (pulmonary thromboembolism, cerebral embolism, deep or superficial vein thrombosis, and systemic embolism), cutaneous (erythematous, livedo reticularis, peripheral or central cyanosis, pigmentation, and macules), neurologic (headache, seizure, vertigo, loss of consciousness, non-cardiac syncope, and psychiatric presentation) and systematic (fatigue, malaise, myalgia, weight loss, thrombocytopenia, anemia, and fever).

### Quality assessment

The methodological quality of the included case reports and case series was assessed using the Mayo Evidence-Based Practice Center tool [18]. The assessment involved evaluating bias in four domains: selection, ascertainment, causality, and reporting. Each question was scored as 0 or 1, resulting in an overall score ranging from 0 to 8. Based on the final score, each study was categorized as having a high risk (≤ 4), moderate risk (5–6), or low risk (≥ 7) of bias. Among these studies, 179 case reports were categorized as high risk, 173 as moderate risk, and 145 studies as low risk.

### Statistical analysis

We performed a quantitatively analyzed using descriptive statistics. Categorical and continuous variables were presented as frequency (percentage) and mean ± standard deviation (SD), respectively. The descriptive analysis was performed using SPSS, version 26.

## Results

### Study selection and characteristics

**Fig. 1:**
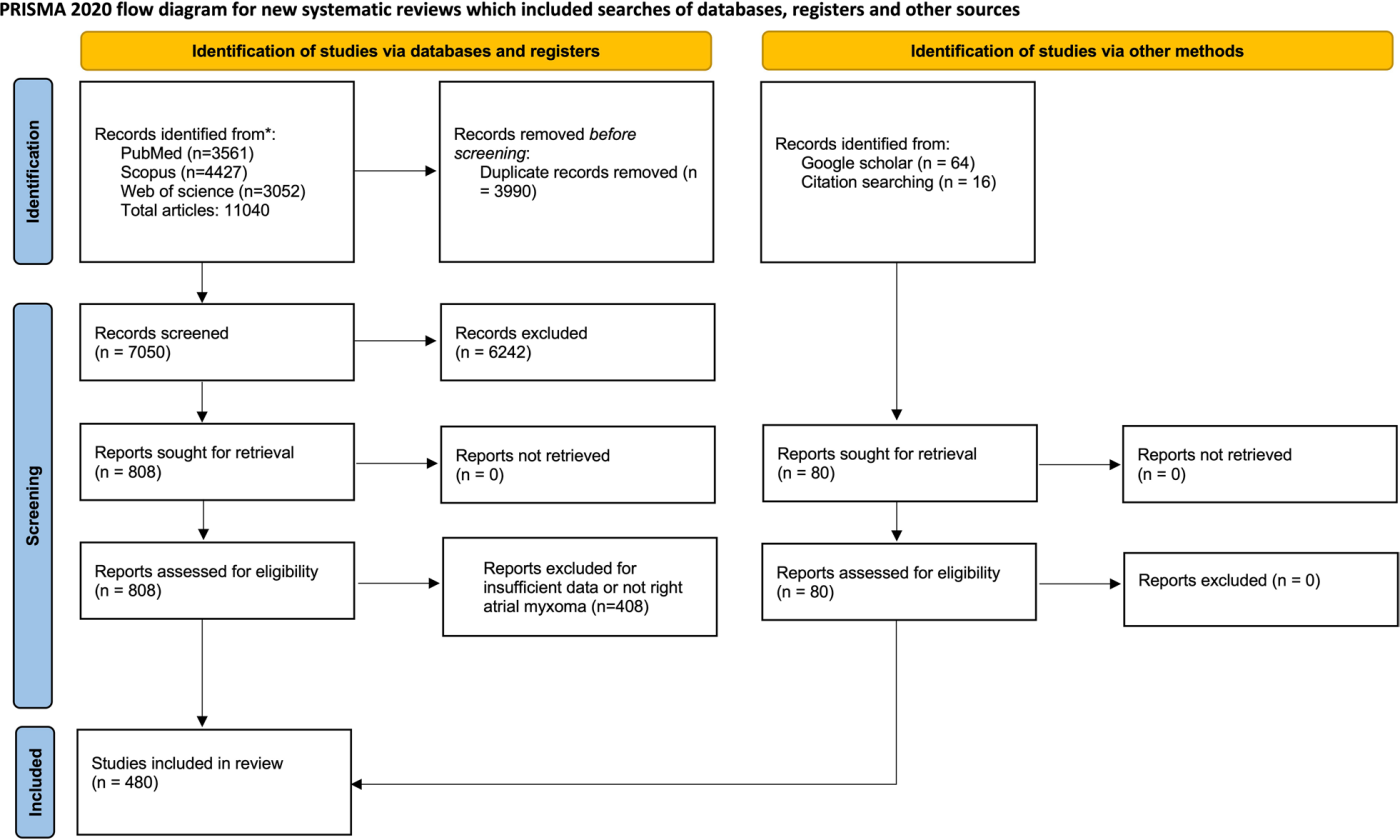
Prisma flow of study selection

**Fig. 2:**
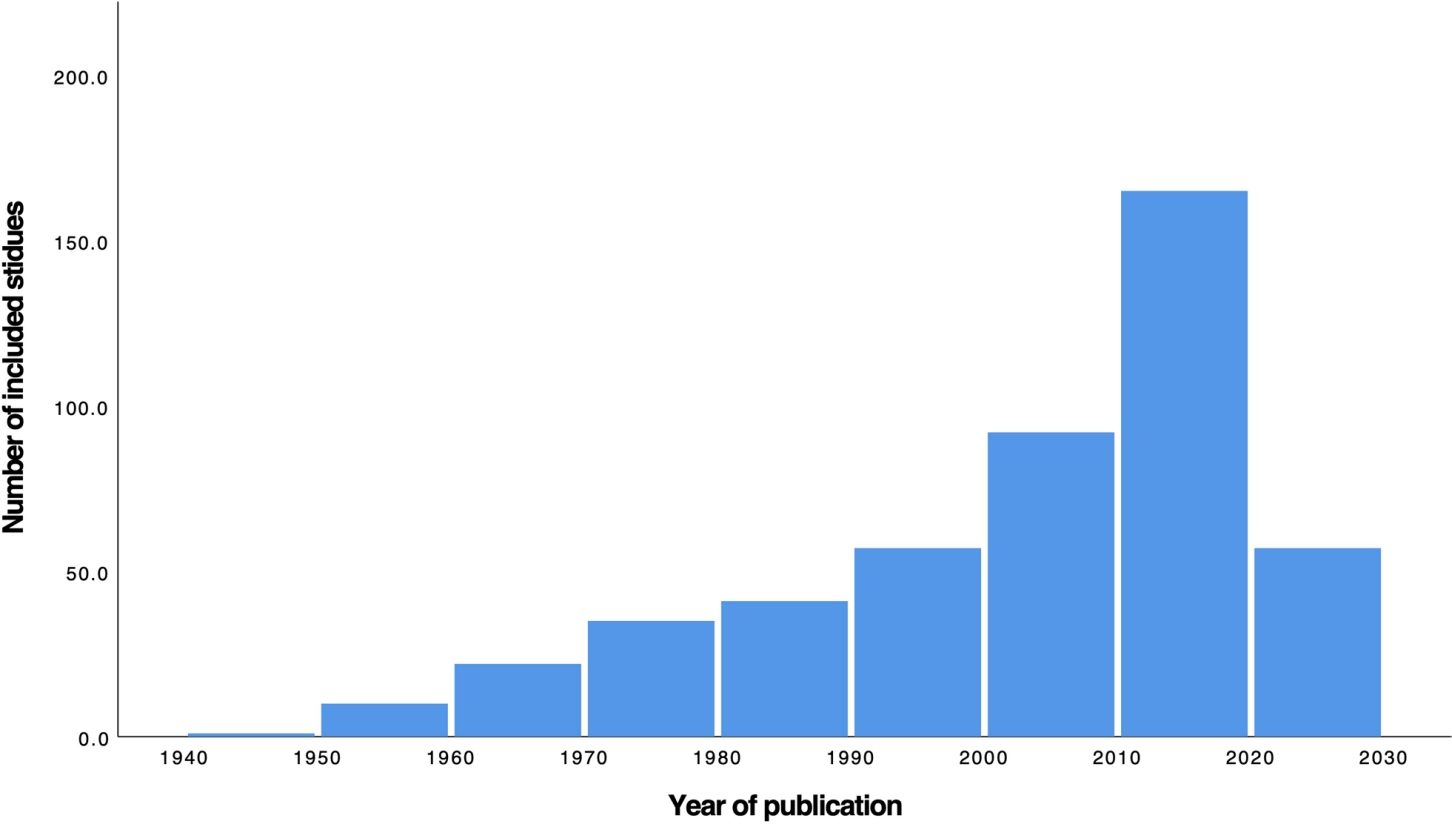
Number of included studies according to the year of publication

**Fig. 3:**
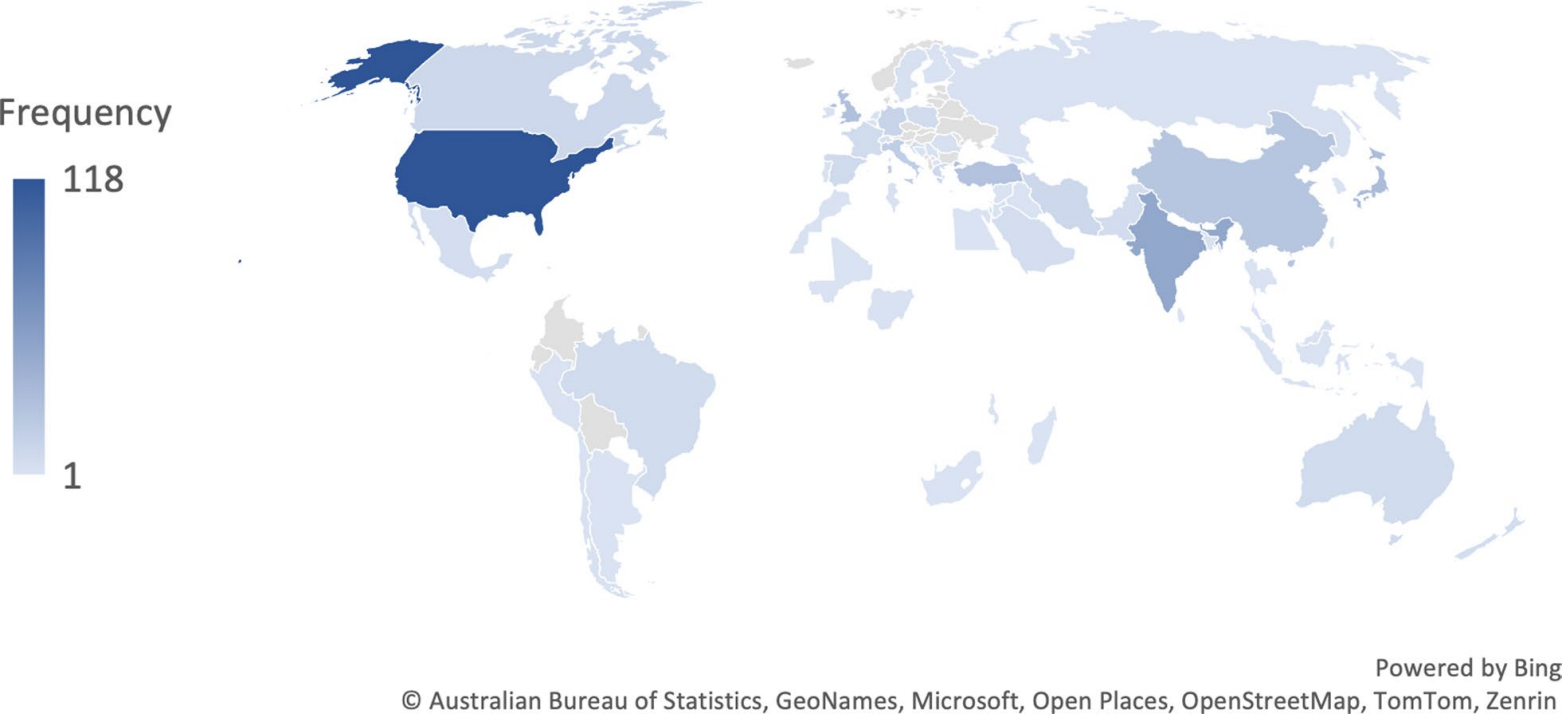
Area-wise distribution of cases with RA myxoma

The first screening identified 11,040 papers in the PubMed, Scopus, and Web of Science. After removing duplicates and excluding based on title abstract screening, 808 record remained for full-text evaluation. Overall, 480 studies included 619 patients met eligible criteria for inclusion in this systematic review (Fig. 1). A summary of included case reports and case series characteristics are presented in Table S1. The year of publication ranged from 1949 to 2022, and studies were originated from 67 countries. The USA accounts for 179 cases (28.9%), followed by India (10.0%) and China (9.0%) (Figs. 2 and 3).

### Patient demographics

The age of cases ranged from early infant to 89 years, with a mean of 45.7 ± 17.6 years, of whom 257 (44.5%) were male. The age distribution of the cases showed that RA myxoma is more prevalent in adult population, with only 39 cases were under 18 years. The most common comorbidity presents in was hypertension (19.2%) followed by diabetes mellitus (7.6%), and other malignancies (6.8%). Among reported studies, 48 patients (7.75%) presented other locations of myxoma.

### Diagnostic methods

Echocardiography was the most common primary imaging method for RA myxoma diagnosis, which performed in 428 (77.8%) patients. Computed tomography (CT) scan (26.6%), angiocardiography (19.7%), and cardiac magnetic resonance imaging (MRI) (16.9%) were other reported diagnostic imaging modalities (Table 1).

**Table 1:** Demographics of reported cases with right atrial myxoma

Characteristics	Value
Age (years), mean (SD)	45.7 (17.6)
Gender	
Male, *n* (%)	257 (44.5)
Comorbidities	
Hypertension, *n* (%)	53 (19.2)
Diabetes mellitus, *n* (%)	21 (7.6)
Cancer, *n* (%)	19 (6.8)
Dyslipidemia, *n* (%)	9 (3.2)
Diagnostic methods	
Echocardiography, *n* (%)	428 (77.8)
Cardiac MRI, *n* (%)	93 (16.9)
CT scan, *n* (%)	120 (26.6)
Angiocardiography, *n* (%)	89 (19.7)
Autopsy, *n* (%)	12 (2.6)
Coexist myxoma, *n* (%)	48 (7.7)
Outcome	
Died, *n* (%)	45 (9.2)

MRI, magnetic resonance imaging; CT, computed tomography

### Clinical manifestations

**Fig. 4:**
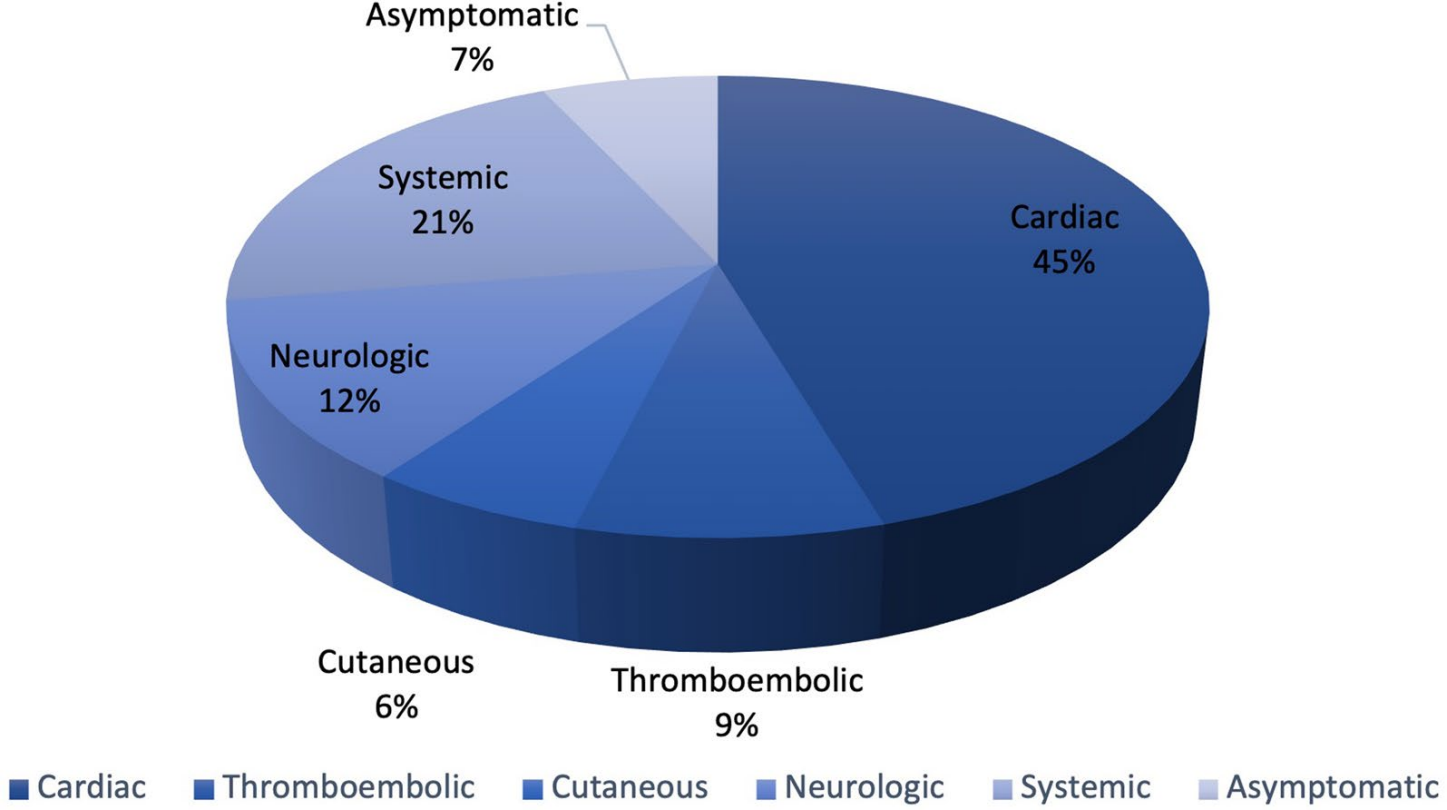
Clinical manifestations of RA myxoma according to system

A wide range of clinical manifestations were reported in the included studies. We divided clinical manifestation into five groups including cardiac, thromboembolic, neurologic, cutaneous, and systemic manifestation. The proportion of each manifestation is presented in Fig. 4. Out the clinical manifestation, cardiac was the most common reported in 477 (77.0%) of cases, followed by systemic manifestation (*n* = 216 (34.8%)), neurologic manifestations (*n* = 131 (21.1%)), and thromboembolic events (*n* = 89 (12.9%)). Besides, 73 (11.7%) cases were diagnosed without any sign and symptoms (Table 2). Syncope was the most common neurological symptom, occurring in 78 cases (59%). Pulmonary embolism was the most frequent thromboembolic event, accounting for 76 cases (85%), followed by multifocal and systemic embolism, which occurred in six cases (6.7%).

**Table 2:** Clinical manifestations of right atrial myxoma

Manifestation	Total, *n* (%)
Neurologic	131 (21.1)
Cutaneous	64 (10.3)
Thromboembolic	89 (12.9)
Cardiac	477 (77.0)
Systemic	216 (34.8)
Asymptomatic	73 (11.7)

### Laboratory findings

Leukocytosis was reported in 44 RA myxoma patients, which identified in 50.0% of reported cases. Moreover, 79.2% and 93.4% of measuring studies showed an increased level of ESR and CRP, respectively. NT-proBNP level was elevated in 12 (92.3%) of patients. Troponin level was elevated only in one (10%) case. All reported cases had elevated INR (Table 3).

**Table 3:** Laboratory findings of patients with right atrial myxoma

Variable	Value, *n* (%)
WBC	
Elevated	44 (50.0)
Normal	44 (50.0)
INR	
Elevated	14 (100)
Normal	0 (0)
ESR	
Elevated	103 (79.2)
Normal	27 (20.8)
CRP	
Elevated	43 (93.4)
Normal	3 (6.6)
Troponin	
Elevated	1 (10)
Normal	9 (90)
NT-proBNP	
Elevated	12 (92.3)
Normal	1 (7.7)
D-dimer	
Elevated	14 (77.8)
Normal	4 (22.2)

WBC, white blood cell; INR, international normalized ratio; ESR, erythrocyte sedimentation rate; CRP, C-reactive protein; NT-proBNP, N-terminal prohormone of brain natriuretic peptide

### Electrocardiogram findings

Out of 337 cases with available ECG data, ECG was normal in 133 (39.4%) of patients. The most commonly reported ECG abnormality were the composite of tall or peaked P-wave, RA and LA enlargement (19.2%), abnormal T-wave (14.0%), sinus tachycardia (11.8%), and incomplete or complete RBBB (11.2%). Besides, ST depression and ST elevation were observed in two and three patients, respectively (Table 4).

**Table 4:** ECG findings and frequencies in the right atrial myxoma cases

ECG	*N* (%)
Normal	133 (39.4)
Incomplete or complete RBBB	38 (11.2)
AF	15 (4.4)
Atrial flutter	6 (1.7)
AV block	7 (1.7)
Tall or peaked P-wave, RA and LA enlargement	65 (19.2)
ST elevation	2 (0.5)
ST depression	3 (0.8)
Low voltage	28 (8.3)
Sinus tachycardia	40 (11.8)
Abnormal T-wave	44 (14.0)
LV hypertrophy	5 (1.4)
RV hypertrophy	12 (3.5)

RBBB, right bundle branch block; AF, atrial fibrillation; AV, Atrioventricular; RA, right atrium; LA, left atrium; LV, left ventricle; RV, right ventricle

### Outcome

A total of 486 cases reported outcome of the patients. Pooled survival rate of these patients was 90.75%. Among the 45 reported deaths, 19 (42.2%) cases were men and 26 (57.73%) cases were women (Table 1). Among these reported deaths, 5 (11.1%) cases were less than 20 years old, 18 (40.0%) cases were between 20 and 40 years old, 16 (35.6%) cases were 40–60 years old and 6 (13.3%) cases were older than 60 years old.

## Discussion

This systematic review summarized the characteristics, outcome, and manifestations of patients with RA myxoma. This study included 480 studies which comprised 619 patients diagnosed with RA myxoma. RA myxoma is a rare condition. Although it may be found in asymptomatic patients, it may be presented with various clinical manifestations including cardiac, systemic, neurological, thromboembolic, and cutaneous symptoms. Increased NT-proBNP and inflammatory markers such as WCB count, CRP, and ESR are reported in patients with RA myxoma. ECG abnormalities were also observed in a significant number of patients, with tall or peaked P-waves, signs of RA and LA enlargement, abnormal T-waves, sinus tachycardia, and RBBB being the most commonly reported findings. The overall survival rate of patients with RA myxoma was high at 90.75%. RA myxoma was uncommon in pediatric cases in this study, with only 39 cases were under 18 years old. Moreover, we found a slight domination by females in RA myxoma patients.

That is in line with previous findings suggestive of a slightly higher prevalence of cardiac myxoma among women [19]. However, these investigations evaluated all cases of cardiac myxoma, whereas our review especially focused on the right atrial myxoma. Additionally, this study identified cardiovascular symptoms as the most common clinical manifestation, which supports our findings that cardiac symptoms are the most prevalent manifestation. However, we have categorized cardiovascular symptoms experienced by patients into cardiac and thromboembolic events. Although cardiac symptoms are more common in these patients, embolization, considering its fetal outcomes and high assurance, needs particular attention.

In this study, we found that 12.9% of the patients with RA myxoma have presented thromboembolic events, which included pulmonary, multifocal, and cerebral embolism. Previous studies also demonstrated atypical location of cardiac myxoma is associated with increased risk of embolic events in these patients [20, 21]. Hence, RA myxoma should be considered in the differential diagnosis of cases with multiple thromboembolic events.

After cardiac symptoms, systemic and neurologic symptoms presented the highest prevalence, respectively. However, a study on left atrial myxoma suggested that constitutional symptoms like fatigue are the least prevalent [22]. Another study suggested a 50% prevalence of constitutional symptoms and 26–45% of neurologic symptoms among left myxoma patients, which are both higher compared to our result [23]. These discrepancies may highlight the potential differences between symptoms of right and left atrial myxoma and warrant further research in this regard.

The amount of death among these cases with RA myxoma was low (9.25%). This suggests that early detection and surgical removal of the tumor can lead to favorable outcomes. However, it is essential to note that delayed diagnosis or complications related to the tumor, such as embolization, may increase the risk of mortality [24–26]. Therefore, prompt diagnosis and timely intervention are crucial in the management of RA myxoma.

RA myxoma may be asymptomatic, and hence, could be found incidentally on imaging modalities. Echocardiography remained the primary diagnostic method for RA myxoma in the majority of cases. Previous studies showed the sensitivity of echocardiography as high as 95% for transthoracic echocardiography (TTE) and 100% for transesophageal echocardiography in detection of cardiac myxoma [27, 28]. TTE was the initial imaging modality used in most cases, followed by TEE for further evaluation. TEE was particularly useful in cases where TTE findings were inconclusive or when there was high clinical suspicion for RA myxoma. Other imaging modalities such as cardiac MRI and CT scan were used in a minority of cases. However, it is important to note that echocardiography remains the gold standard for diagnosing cardiac myxomas.

This is the first systematic review evaluated the clinical, laboratory, and ECG findings of patients with RA myxoma, and provides valuable insights into the clinical characteristics and outcomes of patients with RA myxoma and can contribute to the development of evidence-based management strategies for this rare cardiac tumor. The comprehensive search conducted across prominent databases enhances the reliability of this study. This study has some limitations. Firstly, only case reports and case series were included in this systematic review, introducing a potential risk of bias. Secondly, there were lack of data regarding some the required information in most of the included studies. Thirdly, most of included studies (90.75%) reported positive outcome, hence, publication bias should be considered. Finally, despite efforts to conduct a comprehensive literature search, there is a possibility of missing relevant studies due to limitations in the search strategy or databases used.

## Conclusions

In conclusion, this systematic review provides a comprehensive summary of the clinical and laboratory manifestations and outcomes of RA myxoma, contributing to the existing knowledge on this rare cardiac tumor. RA myxoma presents with a wide range of clinical symptoms, and ECG findings can aid in the diagnosis. Echocardiography remains the mainstay of diagnosis, while early surgical intervention can lead to favorable outcomes. Further research and larger studies are needed to enhance our understanding of this rare cardiac tumor and to improve diagnostic and management strategies.

## Supplementary Information

Supplementary Material 1.

## Data Availability

The datasets used and/or analyzed during the current study are available from the corresponding author on reasonable request.

## Abbreviations

AF: Atrial fibrillation
CRP: C-reactive protein
CT: Computed tomography
ECG: Electrocardiogram
ESR: Erythrocyte sedimentation rate
INR: International normalized ratio
LA: Left atrium
LV: Left ventricle
MRI: Magnetic resonance imaging
NT-proBNP: N-terminal prohormone of brain natriuretic peptide
PRISMA: Preferred reporting items for systematic reviews and meta-analysis
RA: Right atrium
RV: Right ventricle
RBBB: Right bundle branch block
WBC: White blood count
