# Supplementary material for: Clinical and laboratory manifestations, ECG findings, and outcomes of right atrial myxoma: a systematic review of cases reported worldwide
Source: Egypt Heart J. 2024 Sep 12;76:125. doi: 10.1186/s43044-024-00550-x (PMC11393240; doi:10.1186/s43044-024-00550-x)
Supplement: Supplementary file 1 — Supplementary Material 1. [file 43044_2024_550_MOESM1_ESM.docx]

| **Authors** | **Year** | **Country** | **Age** | **Gender** | **Number of patients** | **Outcome** | **Risk of bias score** |
| --- | --- | --- | --- | --- | --- | --- | --- |
| Velvet et al. | 2022 | England | 48 | F | 1 | Alive | Moderate |
| Ma et al. | 2022 | China | 59 | F | 1 | Alive | Moderate |
| He et al. | 2022 | China | 56 | M | 1 | Alive | Low |
| Gong et al. | 2022 | China | 33 | F | 1 | Alive | High |
| Gomase et al. | 2022 | India | 8 days | - | 1 | Alive | Moderate |
| Javeed et al. | 2022 | USA | 70 | M | 1 | Alive | Moderate |
| He et al. | 2022 | China | 44 | M | 1 | Alive | High |
| Straw et al. | 2022 | Germany | 66 | F | 1 | Alive | Low |
| Salehi et al. | 2022 | Iran | 38 | M | 1 | Not reported | High |
| Ran et al. | 2022 | China | 17 | F | 1 | Alive | Low |
| Henmi et al. | 2022 | Italy | 56 | F | 1 | Alive | High |
| Gewehr et al. | 2022 | Brazil | 69 | F | 1 | Alive | Moderate |
| Singh et al. | 2022 | India | 22 | M | 1 | Alive | Moderate |
| Randriamanga et al. | 2022 | Madagascar | 17 | M | 1 | Alive | Low |
| Mielczarek et al. | 2022 | Poland | 42 | F | 1 | Alive | Low |
| Khansari et al. | 2022 | Iran | 47 | M | 1 | Alive | High |
| Alessio et al. | 2022 | Italy | 69 | F | 1 | Alive | High |
| Šaler et al. | 2022 | Croatia | 60 | M | 1 | Alive | Moderate |
| Sanchez-Soteoto et al. | 2022 | Mexico | 59 | M | 1 | Alive | Moderate |
| Naser et al. | 2021 | Bosnia and Herzegovina | 77 | F | 1 | Not reported | Moderate |
| Manda et al. | 2021 | Malawi | 30 | M | 1 | Died | Low |
| Kynta et al. | 2021 | India | 49 | M | 1 | Alive | Low |
| Hsi et al. | 2021 | USA | 38 | F | 1 | Alive | Moderate |
| Garcia et al. | 2021 | Mexico | 27 | M | 1 | Alive | Moderate |
| Altujjar et al. | 2021 | USA | 55 | F | 1 | Alive | Moderate |
| Yamamoto et al. | 2021 | Japan | 72 | F | 1 | Alive | Moderate |
| Forbes et al. | 2021 | USA | 58 | F | 1 | Alive | High |
| Menon et al. | 2021 | India | 39 | M | 1 | Alive | High |
| Eftekharzadeh et al. | 2021 | USA | 61 | F | 1 | Alive | Moderate |
| Disney et al. | 2021 | USA | 46 | F | 1 | Alive | Low |
| Contreras et al. | 2021 | Mexico | 53 | F | 1 | Not reported | High |
| Obagi et al. | 2021 | USA | 30 | F | 1 | Alive | Moderate |
| Alizadehasl et al. | 2021 | Iran | 59 | M | 1 | Not reported | High |
| Saadeh et al. | 2021 | Jordan | 38 | F | 1 | Alive | Low |
| Jayaweera et al. | 2021 | Sri Lanka | 46 | M | 1 | Alive | Low |
| Saha et al. | 2021 | India | 45 | F | 1 | Not reported | High |
| Venkatesan et al. | 2021 | India | 29 | F | 1 | Alive | Moderate |
| Bharath et al. | 2021 | India | 57 | F | 1 | Alive | High |
| Anitha et al. | 2021 | India | 42 | M | 1 | Alive | High |
| Bulić et al. | 2021 | Croatia | 70 | F | 1 | Alive | High |
| Selvaganesan et al. | 2020 | USA | 59 | F | 1 | Not reported | High |
| Papadopoulos et al. | 2020 | USA | 50 | F | 1 | Alive | High |
| Ma et al. | 2020 | China | 51 | F | 1 | Alive | Moderate |
| Garg et al. | 2020 | India | 50 | M | 1 | Not reported | High |
| Elhamamsy et al. | 2020 | Saudi Arabia | 29 | F | 1 | Alive | High |
| Al Sergani et al. | 2020 | Saudi Arabia | 33 | F | 1 | Alive | High |
| Tang et al. | 2020 | China | 72 | F | 1 | Alive | High |
| Lempesis et al. | 2020 | UK | 35 | F | 1 | Alive | Low |
| Agstam et al. | 2020 | India | 37 | M | 1 | Alive | Low |
| Minhas et al. | 2020 | - | 49 | M | 1 | Not reported | High |
| Numaguchi et al. | 2020 | Japan | 59 | F | 1 | Alive | Moderate |
| Tyson et al. | 2020 | UK | 76 | M | 1 | Not reported | High |
| Faisal et al. | 2020 | Pakistan | 18 | F | 1 | Alive | Low |
| Lopez et al. | 2020 | USA | 73 | M | 1 | Alive | Low |
| Vargas et al. | 2020 | Mexico | 29 | F | 1 | Died | High |
| Dindaş et al. | 2020 | Turkey | 72 | F | 1 | Alive | Moderate |
| Shevchenko et al. | 2020 | Russia | 5 | F | 1 | Alive | Moderate |
| Fernandes et al. | 2019 | Florida | 60 | M | 1 | Alive | Low |
| Xiong et al. | 2019 | China | 47 | M | 1 | Alive | High |
| Ma et al. | 2019 | China | 55 | M | 1 | Alive | Low |
| Huang et al. | 2019 | Taiwan | 14 | F | 1 | Not reported | High |
| Bhosle et al. | 2019 | India | 24 | M | 1 | Died | High |
| Nawaz et al. | 2019 | USA | 56 | F | 1 | Not reported | High |
| Dey et al. | 2019 | India | 34 | M | 1 | Alive | High |
| Durand et al. | 2019 | France | 20 | F | 1 | Alive | High |
| Natraj Setty et al. | 2019 | India | 40 | F | 1 | Alive | Low |
| Alamri et al. | 2019 | New Zealand | 53 | M | 1 | Alive | Moderate |
| Beiras-Fernandez et al. | 2019 | Germany | 63 | M | 1 | Alive | Moderate |
| Bhatti et al. | 2019 | USA | 33 | F | 1 | Not reported | High |
| Pandey et al. | 2019 | USA | 32 | F | 1 | Alive | Moderate |
| Peregud-Pogorzelska et al. | 2019 | Poland | 55 | M | 1 | Alive | Moderate |
| Merli et al. | 2019 | Italy | 33 | F | 1 | Alive | Moderate |
| Younes et al. | 2019 | Syria | 4 | M | 1 | Alive | Moderate |
| Al Hussein et al. | 2019 | Romania | 70 | M | 1 | Alive | Moderate |
| Usman et al. | 2019 | Nigeria | 20 | F | 1 | Not reported | High |
| Silva et al. | 2019 | USA | 35 |  | 1 | Alive | Moderate |
| Xie et al. | 2018 | China | 49 | F | 1 | Alive | High |
| Xie et al. | 2018 | China | 4 | F | 1 | Alive | High |
| Sultan et al. | 2018 | Pakistan | 29 | F | 1 | Alive | High |
| Sharifkazemi et al. | 2018 | Iran | 55 | M | 1 | Alive | Moderate |
| Sahitya et al. | 2018 | India | 65 | F | 1 | Not reported | High |
| Longatto et al. | 2018 | Brazil | 55 | F | 1 | Alive | High |
| Hinić et al. | 2018 | Serbia | 62 | M | 1 | Alive | Moderate |
| Haruki et al. | 2018 | Japan | 65 | F | 1 | Not reported | High |
| Agrawal et al. | 2018 | India | 52 | M | 1 | Alive | Moderate |
| Kuroda et al. | 2018 | Japan | 69 | M | 1 | Alive | High |
| Vega Adauy et al. | 2018 | Chile | 59 | F | 1 | Not reported | High |
| Changal et al. | 2018 | USA | 56 | F | 1 | Alive | High |
| Flint et al. | 2018 | USA | 60 | M | 1 | Alive | Low |
| Jia et al. | 2018 | China | 29 | M | 1 | Alive | Moderate |
| Munirathinam et al. | 2018 | India | 24 | M | 1 | Alive | High |
| Sanjeev et al. | 2018 | India | 32 | F | 1 | Alive | Moderate |
| Choi et al. | 2018 | UK | 72 | F | 1 | Alive | High |
| Chen et al. | 2018 | China | 18 months | M | 1 | Alive | Moderate |
| Heidari et al. | 2018 | Iran | 41 | F | 1 | Alive | Moderate |
| Mudgalkar et al. | 2018 | India | 60 | F | 1 | Alive | Moderate |
| Yanardag et al. | 2018 | Turkey | 18 | M | 1 | sudden death | Moderate |
| Cervetti et al. | 2018 | Argentina | 42 | F | 1 | Not reported | High |
| Lee et al. | 2017 | Taiwan | 77 | F | 1 | Alive | High |
| Sinha et al. | 2017 | India | 42 | M | 1 | Alive | Moderate |
| Pehlivan et al. | 2017 | Turkey | 43 | F | 1 | Died | High |
| Nivargi et al. | 2017 | India | 34 | M | 1 | Not reported | High |
| Hamid et al. | 2017 | Malaysia | 32 | F | 1 | Died | Low |
| Bandyopadhyay et al. | 2017 | India | 40 | F | 1 | Alive | High |
| Nath et al. | 2017 | India | 35 | M | 1 | Alive | Moderate |
| Rathor et al. | 2017 | India | 52 | M | 1 | Alive | Moderate |
| Abdallah e al. | 2017 | Saudi Arabia | 55 | 4M/8F | 12 | Alive: 12 (100%) | Moderate |
| Sheriff et al. | 2017 | India | 42 | F | 1 | Alive | Moderate |
| Singhal et al. | 2017 | India | 35 | F | 1 | Alive | Moderate |
| Srivastava et al. | 2017 | India | 48 | M | 1 | Alive | High |
| Kumar et al. | 2017 | India | 37 | F | 1 | Alive | Moderate |
| Mohammad Karimi et al. | 2016 | Iran | 71 | F | 1 | Not reported | Moderate |
| Majumdar et al. | 2016 | India | 36 | F | 1 | Alive | Moderate |
| Lewitowicz et al. | 2016 | Poland |  |  | 7 | Not reported | High |
| Kwon et al. | 2016 | Korea | 46 | F | 1 | Alive | High |
| Ghasemi et al. | 2016 | Iran | 45 | M | 1 | Alive | Moderate |
| Essandoh et al. | 2016 | USA | 50 | M | 1 | Alive | Moderate |
| Dong et al. | 2016 | China | 48 | F | 1 | Alive | High |
| Liu et al. | 2016 | China | 39 | F | 5 | Alive | Moderate |
|  |  |  | 52 | F |  | Alive |  |
|  |  |  | 49 | M |  | Alive |  |
|  |  |  | 44 | M |  | Alive |  |
|  |  |  | 44 | M |  | Alive |  |
| Molnar et al. | 2016 | Romania | 73 | F | 1 | Not reported | High |
| Yin et al. | 2016 | China | 34 | M | 1 | Not reported | Moderate |
| Santos-Ortega et al. | 2016 | Spain | 57 | F | 1 | Alive | Low |
| Muthiah et al. | 2016 | India | 12 | M | 1 | Died | Low |
| Sangodkar et al. | 2016 | USA | 67 | M | 1 | Not reported | High |
| Nakabayashi et al. | 2016 | Japan | 64 | F | 1 | Alive | Moderate |
| Sato et al. | 2016 | Japan | 70 | M | 1 | Alive | High |
| Menti et al. | 2016 | Brazil | 37 | F | 1 | Alive | Moderate |
| Goksuluk et al. | 2016 | Turkey | 37 | M | 1 | Alive | Moderate |
| Oumar et al. | 2016 | Mali | 52 | M | 1 | Alive | Moderate |
| Singh et al. | 2016 | India | 26 | F | 1 | Alive | Low |
| Ungerman et al. | 2016 | USA | 72 | M | 1 | Alive | High |
| Zairi et al. | 2015 | Tunisia | 44 | F | 1 | Alive | High |
| Şaşkın et al. | 2015 | Turkey | 62 | M | 1 | Alive | Low |
| Salehi et al. | 2015 | Iran | 57 | M | 1 | Alive | High |
| Kumary et al. | 2015 | India | 38 | F | 1 | Alive | High |
| Guo et al. | 2015 | China | 22 | M | 1 | Alive | High |
| Gromadziński et al. | 2015 | Poland | 51 | F | 1 | Alive |  |
| Animashaun et al. | 2015 | USA | 89 | M | 1 | Not reported | High |
| Rokadia et al. | 2015 | USA | 33 | M | 1 | Alive | High |
| Abdelaziz et al. | 2015 | Egypt | 43 | F | 1 | Alive | High |
| Romanović et al. | 2015 | Serbia | 77 | F | 1 | Alive | Low |
| Pussadhamma et al. | 2015 | Thailand | 52 | F | 1 | Not reported | High |
| Kurnicka et al. | 2015 | Poland | 62 | F | 1 | Not reported | High |
| Li et al. | 2015 | China | 47.77 | 16M/12F | 28 | Alive: 28 (100%) | Moderate |
| Ikeda et al. | 2014 | Japan | 74 | M | 1 | Alive | Moderate |
| Gu et al. | 2014 | China | 33 | M | 1 | Alive | Moderate |
| Dharmalingam et al. | 2014 | India | 43 | M | 1 | Alive | High |
| Bhalerao et al. | 2014 | India | 22 | M | 1 | Alive | High |
| Arnáiz-García et al. | 2014 | Spain | 45 | F | 1 | Not reported | High |
| Min et al. | 2014 | South Korea | 36 | F | 1 | Alive | Moderate |
| Strecker et al. | 2014 | Germany | 62 | F | 1 | Alive | Moderate |
| Bezgin et al. | 2014 | Turkey | 18 | F | 1 | Alive | Moderate |
| Konency et al. | 2014 | UK | 46 | M | 1 | Alive | Moderate |
| Dorobantu et al. | 2014 | Romania | 47 | M | 1 | Alive | High |
| Kaya et al. | 2014 | Malatya | 29 | M | 1 | Alive | Low |
| Jovanova et al. | 2014 | Macedonia | 67 | M | 1 | Alive | Moderate |
| Barakizou et al. | 2014 | Tunisia | 4 | F | 1 | Alive | Low |
| Sharma et al. | 2013 | UK | 21 | M | 1 | Alive | Moderate |
| Tataroǧlu et al. | 2013 | Turkey | 71 | F | 1 | Not reported | High |
| Sansone et al. | 2013 | Italy | 74 | F | 2 | Alive | High |
|  |  |  | 58 | M |  | Alive |  |
| Mehrotra et al. | 2013 | New Zealand | 64 | M | 1 | Alive | High |
| Liu et al. | 2013 | China | 48 | F | 1 | Not reported | High |
| Jung et al. | 2013 | Korea | 76 | F | 1 | Alive | Low |
| Atipo-Galloye et al. | 2013 | Morocco | 32 | F | 1 | Alive | High |
| Nazzi et al. | 2013 | France | 60 | M | 1 | Alive | Moderate |
| Marinakis et al. | 2013 | Belgium | 78 | F | 1 | Alive | Low |
| Berger et al. | 2013 | Switzerland | 61 | F | 1 | Died | High |
| Kumar et al. | 2013 | India | 48 | M | 1 | Alive | High |
| Wang et al. | 2013 | Australia | 56 | F | 1 | Not reported | High |
| Kontogiorgi et al. | 2013 | Greece | 28 | M | 1 | Alive | Moderate |
| Omar et al. | 2013 | USA | 82 | F | 1 | Alive | Low |
| Xiao et al. | 2013 | China | 59 | F | 1 | Alive | Moderate |
| Carvalho et al. | 2013 | Portugal | 35 | M | 2 | Alive | High |
|  |  |  | 38 |  |  | Alive |  |
| Aydın et al. | 2013 | Turkey | 29 | M | 1 | Alive | Moderate |
| Batinić et al. | 2013 | Croatia | 63 | M | 1 | Alive | Low |
| Kumpare et al. | 2013 | Croatia | 70 | F | 1 | Alive | High |
| Subban et al. | 2012 | India | 38 | F | 1 | Alive | High |
| Shamim et al. | 2012 | UK | 50 | F | 1 | Alive | Moderate |
| Oommen et al. | 2012 | Brunei | 55 | M | 1 | Alive | Moderate |
| Nina et al. | 2012 | Brazil | 45 | F | 1 | Alive | Moderate |
| Kale et al. | 2012 | India | 15 | M | 2 | Alive | High |
|  |  |  | 42 | M |  | Alive |  |
| Horne et al | 2012 | Canada | 60 | F | 1 | Not reported | High |
| Hatz et al. | 2012 | USA | 47 | F | 2 | Alive | High |
|  |  |  | 58 | F |  | Alive |  |
| Chhabra et al. | 2012 | USA | 72 | F | 1 | Alive | Moderate |
| Çakmak Gökçe et al. | 2012 | Turkey | 39 | M | 1 | Alive | High |
| Cheema et al. | 2012 | USA | 57 | M | 1 | Alive | High |
| Rao et al. | 2012 | India | 18 | M | 1 | Alive | Moderate |
| Demirtürk et al. | 2012 | Turkey | 21 | M | 1 | Alive | Moderate |
| Dobson et al. | 2012 | UK | 29 | M | 1 | Alive | Moderate |
| Romeo et al. | 2012 | Italy | 72 | F | 1 | Alive | Low |
| Sato et al. | 2012 | Japan | 75 | F | 1 | Alive | Low |
| Acikel et al. | 2012 | Turkey | 46 | F | 1 | Alive | Low |
| Sabageh et al. | 2012 | Nigeria | 50 | M | 1 | Sudden death | Moderate |
| Boutayeb et al. | 2012 | Morocco | 57 | F | 1 | Alive | Moderate |
| Sabzi et al. | 2012 | Iran | 24 | M | 1 | Alive | Moderate |
| Yeshvanth et al. | 2011 | India | 55 | M | 1 | Not reported | High |
| Shetty Roy et al. | 2011 | USA | 38 | F | 1 | Alive | Moderate |
| Onan et al. | 2011 | Turkey | 41 | M | 1 | Alive | Low |
| Han et al. | 2011 | China | 42 | F | 1 | Alive | High |
| Alizade et al. | 2011 | India | 84 | F | 1 | Alive | Moderate |
| Manduz et al. | 2011 | Turkey | 35 | F | 3 | Alive | Moderate |
|  |  |  | 60 | F |  | Alive |  |
|  |  |  | 62 | F |  | Alive |  |
| Yang et al. | 2011 | Taiwan | 65 | M | 1 | Alive | Moderate |
| Fatimi et al. | 2011 | Pakistan | 64 | M | 1 | Alive | Low |
| Darwazah et al. | 2011 | Israel | 26 | F | 1 | Alive | Moderate |
| Gogas et al. | 2011 | Greece | 45 | F | 1 | Alive | Moderate |
| Weiss et al. | 2011 | USA | 14 | M | 1 | Alive | Moderate |
| Jara-Palomares et al. | 2011 | Spain | 28 | F | 1 | Not reported | High |
| Mallick et al. | 2010 | India | 10 | M | 1 | Not reported | High |
| Leonard et al. | 2010 | Ireland | 53 | F | 1 | Alive | Moderate |
| Fujiwara et al. | 2010 | Japan | 75 | F | 1 | Alive | High |
| Rafiq et al. | 2010 | UK | 73 | M | 1 | Not reported | High |
| Naqshband et al. | 2010 | Pakistan | 22 | F | 1 | Alive | Moderate |
| Gopalakrishnan et al. | 2010 | India | 55 | M | 1 | Alive | High |
| Ridge et al. | 2010 | Ireland | 53 | F | 1 | Alive | Moderate |
| Sahin et al. | 2010 | Turkey | 48 | F | 1 | Alive | High |
| Azevedo et al. | 2010 | Portugal | 74 | F | 1 | Alive | Moderate |
| Funk et al. | 2010 | USA | 60 | F | 1 | Alive | Moderate |
| Affronti et al. | 2010 | Italy | 57 | M | 1 | Alive | High |
| Karayannis et al. | 2010 | Greece | 78 | F | 1 | Alive | High |
| Vohra et al. | 2010 | UK | 38 | M | 1 | Alive | Moderate |
| Korkmaz et al. | 2010 | Turkey | 20 | M | 1 | Alive | High |
| Tagawa et al. | 2010 | Japan | 68 | M | 1 | Alive | High |
| Yavari et al. | 2009 | UK | 57 | M | 1 | Alive | Moderate |
| Ozer et al | 2009 | Turkey | 58 | F | 1 | Alive | High |
| Modi et al. | 2009 | USA | 25 | F | 1 | Died | High |
| Low et al. | 2009 | Singapore | 47 | F | 1 | Alive | High |
| Chamsi-Pasha et al. | 2009 | Saudi Arabia | 40 | F | 1 | Alive | Moderate |
| Canale et al. | 2009 | Brazil | 43 | F | 1 | Alive | High |
| Ancona et al. | 2009 | Italy | 55 | M | 1 | Not reported | High |
| Charokopos et al. | 2009 | Greece | 65 | F | 1 | Alive | High |
| Haime et al. | 2009 | USA | 57 | M | 1 | Alive | High |
| Pliakos et al. | 2009 | Greece | 28 | F | 1 | Alive | Low |
| Fracasso et al. | 2009 | Germany | 35 | F | 1 | Sudden cardiac death | Low |
| Yuan et al. | 2009 | Israel | 43 | M | 1 | Alive | High |
| Quashem et al. | 2009 | Bangladesh | 45 | F | 1 | Alive | Moderate |
| Chu et al. | 2009 | Australia | 34 | M | 1 |  | Low |
| Tondelli et al. | 2008 | Italy | 49 | F | 1 | Alive | High |
| Scohy et al. | 2008 | Netherlands | 59 | F | 1 | Not reported | High |
| Patrianakos et al. | 2008 | Greece | 67 | M | 1 | Not reported | High |
| Ojji et al. | 2008 | Australia | 51 | M | 1 | Died | Moderate |
| Lorentz et al. | 2008 | Brazil | 42 | M | 1 | Alive | High |
| Bilku et al. | 2008 | UK | 39 | F | 1 | Alive | Moderate |
| Ahmed et al. | 2008 | UK | 45 | F | 1 | Alive | High |
| Ibrahim et al. | 2008 | USA | 51 | M | 1 | Alive | Moderate |
| Shaikh et al. | 2008 | Pakistan | 32 | F | 1 | Alive | Low |
| Cappell et al. | 2008 | USA | 52 | M | 1 | Alive | Moderate |
| Rajani et al. | 2008 | UK | 69 | F | 1 | Alive | Moderate |
| Fayad et al. | 2008 | France | 67 | F | 1 | Not reported | High |
| Sato et al. | 2008 | Japan | 21 | M | 1 | Sudden cardiac death | Moderate |
| Siraj et al. | 2008 | Bangladesh | 56 | M | 1 | Not reported | High |
| Dewi et al. | 2008 | Indonesia | 11 | F | 1 | Not reported | Moderate |
| Schwab et al. | 2007 | Germany | 72 | M | 1 | Not reported | High |
| Hayek et al. | 2007 | USA | 79 | F | 1 | Alive | Low |
| Fang et al. | 2007 | USA | 35 | F | 1 | Alive | Moderate |
| Barlis et al. | 2007 | Australia | 46 | M | 1 | Alive | Low |
| Yanagawa et al. | 2007 | Japan | 51 | F | 1 | Alive | Low |
| Yuce et al. | 2007 | Turkey | 56 | M | 1 | Alive | High |
| Tok et al. | 2007 | Turkey | 52 | M | 1 | Alive | Low |
| Mendoza et al. | 2007 | Peru | 77 | M | 1 | Not reported | High |
| Attili et al. | 2007 | Germany | 66 | M | 1 | Not reported | High |
| Umeda et al. | 2007 | Japan | 60 | M | 1 | Alive | Moderate |
| Kapusta et al. | 2007 | Poland | 62 | F | 1 | Alive | Low |
| Lamarche et al. | 2007 | Canada | 58 | F | 1 | Alive | High |
| Lin et al. | 2007 | China | 65 | F | 1 | Alive | Low |
| Ragland et al. | 2006 | USA | 47 | F | 1 | Alive | High |
| Nakamura et al. | 2006 | Japan | 55 | F | 1 | Alive | High |
| Kumar et al. | 2006 | India | 76 | F | 1 | Not reported | High |
| Jayaprasad et al. | 2006 | India | 50 | M | 1 | Not reported | High |
| Carrasco et al. | 2006 | Chile | 34 | F | 1 | Not reported | High |
| Karlof et al. | 2006 | USA | 58 | M | 1 | Not reported | High |
| Banerjee et al. | 2006 | UK | 75 | M | 1 | Sudden cardiac Died before surgery | Moderate |
| Fabijanic et al. | 2006 | Croatia | 47 | M | 1 | Alive | Low |
| Gupta et al. | 2006 | USA | 65 | M | 1 | Alive | High |
| Yilmaz et al. | 2006 | Turkey | 37 | M | 1 | Alive | Moderate |
| Reynen et al. | 2006 | Germany | 63 | M | 1 | Not reported | High |
| Al Ani et al. | 2006 | Iraq | 45 | M | 1 | Alive | Moderate |
| Filho et al. | 2006 | Brazil | 67 | F | 1 | Alive | Low |
| Göz et al. | 2006 | Turkey | 57 | M | 1 | Alive | Moderate |
| Pillai et al. | 2005 | Canada | 21 | F | 1 | Alive | High |
| Niarchos et al. | 2005 | Greece | 57 | M | 1 | Alive | Low |
| Mittle et al. | 2005 | USA | 35 | F | 1 | Not reported | High |
| Miller et al. | 2005 | USA | 69 | M | 1 | Alive | High |
| Imai et al. | 2005 | Japan | 60 | M | 1 | Alive | Low |
| Riad et al. | 2005 | USA | 36 | M | 1 | Alive | Low |
| Kollias et al. | 2004 | UK | 71 | M | 1 | Alive | Low |
| Aoyagi et al. | 2004 | Japan | 62 | F | 1 | Alive | High |
| Skamrov et al. | 2004 | Russia | 59 | F | 1 | Alive | High |
| Sugeng et al. | 2004 | USA | 66 | F | 1 | Not reported | High |
| Anagnostopoulos et al. | 2004 | Greece | 52 | M | 1 | Alive | Low |
| Yazici et al. | 2004 | Turkey | 65 | F | 1 | Alive | Moderate |
| Kuon et al. | 2004 | Germany | 68 | M | 2 | Not reported | High |
|  |  |  | 70 | F |  | Not reported |  |
| Kejriwal et al. | 2003 | Australia | 53 | M | 1 | Alive | Moderate |
| Eckhardt et al. | 2003 | Switzerland | 61 | F | 1 | Died | Moderate |
| Battellini et al. | 2003 | Germany | 65 | F | 1 | Alive | Moderate |
| Kuralay et al. | 2003 | Turkey | 21 | M | 1 | Alive | Low |
| Hemachandrana et al. | 2003 | India | 50 | F | 1 | Not reported | High |
| Parsons et al. | 2003 | USA | 22 | F | 1 | Alive | Moderate |
| Grebenc et al. | 2002 | USA | - | - | 23 | Not reported | High |
|  |  |  | - | - | 2 | Not reported |  |
| Fácila-Rubio et al. | 2002 | Spain | 25 | F | 1 | Alive | High |
| Sajja et al. | 2001 | India | 74 | M | 1 | Alive | Moderate |
| Puvaneswary et al. | 2001 | Australia | 59 | M | 1 | Not reported | Moderate |
| Oshiumi et al. | 2001 | Japan | 25 | F | 1 | Alive | Low |
| Murayama et al. | 2001 | Japan | 31 | F | 1 | Alive | Low |
| Iacobellis et al. | 2001 | - | - | M | 1 | Not reported | High |
| Mendoza et al. | 2001 | Peru | 15 | F | 1 | Alive |  |
| Reber et al. | 2001 | Germany | 15 | F | 1 | Alive | Moderate |
| Surabhi et al. | 2001 | USA | 77 | F | 1 | Alive | High |
| Alsafwah et al. | 2001 | USA | 30 | F | 1 | Alive | Low |
| Suzuki et al. | 2000 | Japan | 61 | M | 2 | Not reported | High |
|  |  |  | 36 | F |  | Not reported |  |
| Ayan et al. | 2000 | Turkey | 65 | Not reported | 1 | Not reported | High |
| Idir et al. | 2000 | France | 27 | M | 1 | Alive |  |
| Guhathakurta et al. | 2000 | New Zealand | 51 | M | 1 | Alive | Moderate |
| McCoskey et al. | 2000 | USA | 36 | F | 1 | Not reported | Moderate |
| Nardi et al. | 2000 | Italy | 47 | F | 1 | Not reported | High |
| Selvaraj et al. | 1999 | India | 36.7 | 4M/3F | 7 | Alive: 4 (57.1%)  Died:1 (14.3%) | Low |
| Birincioglu et al. | 1999 | Turkey | 13 | F | 1 | Alive | Low |
| Agostini et al. | 1999 | France | 57 | M | 1 | Alive | High |
| Umana et al. | 1999 | USA | 47 | M | 1 | Alive | Moderate |
| Birincioglu et al. | 1999 | Turkey | 13 | F | 1 | Alive | Moderate |
| Moriuchi et al. | 1998 | Japan | 39 | M | 1 | Alive | Moderate |
| Cavero et al. | 1998 | Spain | 46 | M | 1 | Died | Moderate |
| Bitner et al. | 1998 | Poland | 17 | M | 1 | Alive | Low |
| Basso et al. | 1997 | Italy | 66 | F | 1 | Not reported | High |
| Baronciani et al. | 1997 | Italy | 17 | M | 1 | Alive | High |
| Jardine et al. | 1997 | New Zealand | 69 | M | 1 | Died | Low |
| Vassiliadis et al. | 1997 | Greece |  |  |  | Sudden cardiac death | Moderate |
| Goldberg et al. | 1997 | USA | early infancy | F | 1 | Alive | High |
| Marks et al. | 1996 | USA | - | - | 1 | Alive | High |
| Hendricksen et al. | 1996 | USA | 55 | M | 1 | Alive | Low |
| Walker et al. | 1996 | UK | 59 | M | 1 | Alive | Moderate |
| Layfield et al. | 1996 | USA | 20 | M | 1 | Died | Moderate |
| Giacalone et al. | 1996 | Italy | 67 | M | 1 | Died | High |
| Zeppellini et al. | 1995 | Italy | 85 | F | 1 | Alive | Moderate |
| Shimizu et al | 1995 | Japan | 54 | M | 1 | Alive | Moderate |
| Kuroda et al. | 1995 | Japan | 71 | M | 1 | Alive | High |
| Vilacosta et al. | 1994 | Spain | 30 | F | 1 | Alive | Low |
| Tatebe et al. | 1994 | Japan | 24 | F | 1 | Alive | Moderate |
| Suzuki et al. | 1994 | Japan | 13 | M | 1 | Alive | Moderate |
| De Carli et al. | 1994 | Italy | 24 | M | 1 | Alive | Low |
| Richkind et al. | 1994 | USA | 50 | F | 1 | Alive | High |
| Deshpandea et al. | 1994 | India | 18 | F | 2 | Not reported | High |
|  |  |  | 53 | F |  | Not reported |  |
| Kaplan et al. | 1994 | USA | 48 | M | 1 | Alive | Low |
| Saitoh et al. | 1993 | Japan | 61 | M | 1 | Alive | Low |
| Sommariva et al. | 1993 | Italy | 58 | M | 1 | Not reported | Moderate |
| Ohshima et al. | 1993 | Japan | 72 | F | 1 | Not reported | High |
| Nakayama et al. | 1993 | Japan | 39 | M | 1 | Not reported | Moderate |
| Burke et al. | 1993 | USA |  | 9M/9F | 18 | Not reported | High |
| Yen et al. | 1992 | USA | 22 | F | 1 | Not reported | High |
| Nolan et al. | 1992 | Scotland | 74 | M | 1 | Not reported | High |
| Miyauchi et al. | 1992 | Japan | 44 | M | 1 | Alive | Moderate |
| Heck et al. | 1992 | Georgia | 18 | F | 1 | Alive | Low |
| Fox et al. | 1992 | USA | 58 | F | 1 | Alive | Moderate |
| Bakri et al. | 1992 | Saudi Arabia | 36 | F | 1 | Alive | Low |
| De Peppo et al. | 1992 | Italy | 56 | M | 1 | Alive | Moderate |
| Wiedermann et al. | 1992 | Germany | - | - | 1 | Not reported | High |
| Storch et al. | 1991 | USA | 42 | M | 1 | Alive | High |
| Smith et al. | 1991 | USA | 51 | M | 1 | Alive | Moderate |
| Rey et al. | 1991 | Spain | 46 | F | 1 | Not reported | High |
| Lyons et al. | 1991 | USA | 56 | M | 1 | Alive | Moderate |
| Hwang et al. | 1991 | China | 38 | F | 1 | Alive | Low |
| Grover et al. | 1991 | India | 17 | M | 2 | Alive | High |
|  |  |  | 38 | M |  | Alive |  |
| Alam et al. | 1991 | USA | 46 | M | 1 | Not reported | High |
| Turlapati et al. | 1990 | USA | 72 | F | 1 | Alive | Moderate |
| Sellke et al. | 1990 | USA | 52 | F | 5 | Died (colon CA) | High |
|  |  |  | 16 | F |  | Alive |  |
|  |  |  | 64 | F |  | Alive |  |
|  |  |  | 26 | F |  | Alive |  |
|  |  |  | 31 | F |  | Alive |  |
| Roberts et al. | 1990 | UK | 68 | M | 1 | Alive | Moderate |
| Park et al. | 1990 | USA | 5 | M | 1 | Alive | Low |
| Fagan et al. | 1990 | USA | 59 | F | 1 | Alive | High |
| Coughlin et al. | 1990 | Hawaii | 15 | F | 1 | Alive | Low |
| Bortolotti et al. | 1990 | Italy | 41 | 1M/5F | 6 | 1 Died of bowel carcinoma | Moderate |
| Bortolotti et al. | 1990 | Italy | 33 | F | 1 | Alive | Moderate |
| Pasaoglu et al. | 1990 | Turkey | 35 days | M | 1 | Alive | Moderate |
| Umezawa et al. | 1989 | Japan | 72 | F | 1 | Alive | Moderate |
| Nikyar et al. | 1989 | Iran | 5 | M | 1 | Alive | Moderate |
| Moritz et al. | 1989 | USA | 67 | M | 1 | Alive | Moderate |
| Keren et al. | 1989 | Israel | 38 | F | 1 | Alive | Moderate |
| Göksel et al. | 1989 | Turkey | 21 | F | 1 | Alive | Moderate |
| de Roos et al. | 1989 | Netherlands | 64 | F | 1 | Not reported | High |
| Schmid et al. | 1988 | Germany | 66 | F | 1 | Alive | High |
| Rajani et al. | 1988 | India | 40 | F | 3 | Alive | Moderate |
|  |  |  | 40 | F |  | Alive |  |
|  |  |  | 16 | M |  | Alive |  |
| Knepper et al. | 1988 | USA | 31 | F | 1 | Alive | High |
| Jamidar et al. | 1988 | UK | 16 | M | 1 | Alive | Low |
| Milgalter et al. | 1987 | Israel | 75 | M | 2 | Alive | Moderate |
|  |  |  | 34 | F |  | Alive |  |
| Martin et al. | 1987 | USA | 20 | F | 1 | Alive | High |
| Komsuoǧlu et al. | 1987 | Turkey | 55 | F | 1 | Not reported | High |
| Gilkey et al. | 1987 | USA | 71 | F | 1 | Not reported | High |
| Depelchin et al. | 1987 | Belgium | 13 | M | 1 | Not reported | High |
| Cujec et al. | 1987 | Canada | 25 | M | 1 | Alive | Low |
| Wilsher et al. | 1986 | New Zealand | 21 | F | 1 | Died of cardiac arrest after another operation for LA myxoma 8 years later | Moderate |
| Ramsdale et al. | 1986 | UK | 40 | F | 1 | Alive | Low |
| Levin et al. | 1986 | USA | 53 | M | 1 | Alive | High |
| Butler et al. | 1986 | USA | 31 | F | 1 | Alive | Low |
| Seifert et al. | 1986 | USA | 69 | F | 1 | Alive | High |
| Northcote et al. | 1985 | Scotland | 51 | F | 1 | Alive | Low |
| Keller et al. | 1985 | Germany | 20 | M | 1 | Alive | Moderate |
| Fyke et al. | 1985 | USA | 20 | F | 3 | Alive | Moderate |
|  |  |  | 67 | F |  | Alive |  |
|  |  |  | 54 | F |  | Alive |  |
| Pores et al. | 1984 | USA | 60 | M | 1 | Alive | Low |
| Marvasti et al. | 1984 | USA | 16 | M |  | Alive | Moderate |
| Nili et al. | 1983 | Israel | 57 | F | 1 | Alive | Moderate |
| McGarry et al. | 1983 | Canada | 60 | M | 3 | Alive | Low |
|  |  |  | 53 | F |  | Died |  |
|  |  |  | 48 | M |  | Alive |  |
| Gladden et al. | 1983 | USA | 25 | F | 1 | Alive | High |
| Natarajan et al. | 1982 | USA | 63 | F | 1 | Alive | Low |
| Keenan et al. | 1982 | Northern Ireland | 52 | F | 1 | Alive | Moderate |
| Burns et al. | 1982 | USA | 44 | F | 1 | Alive | Low |
| Zheng et al. | 1982 | China | - |  | 1 | Alive | High |
| Dianzumba et al. | 1982 | Jamaica | newly born | F | 1 | Died | Moderate |
| Stern et al. | 1981 | USA | 45 | F | 2 | Alive | High |
|  |  |  | 40 | F |  | Alive |  |
| Willey et al. | 1980 | Scotland | 28 | F | 1 | Alive | Low |
| Oldershaw et al. | 1980 | UK | 24 | M | 1 | Alive | High |
| Atherton et al. | 1980 | UK | 10 | M | 1 | Alive | faily |
| Devig et al. | 1980 | USA | 28 | M | 1 | Alive | Moderate |
| Faruqui et al. | 1980 | Pakistan | 45 | F | 1 | Alive | Low |
| Roudaut et al. | 1980 | France | 7 | M | 1 | Alive | Low |
| Powers et al. | 1979 | USA | 48 | M | 1 | Alive | Low |
| Mori et al. | 1979 | Japan | 48 | F | 1 | Alive | Low |
| Martinez et al. | 1979 | Mexico | 12 | F | 1 | Alive | Moderate |
| Frishman et al. | 1979 | USA | 57 | F | 1 | Alive | Low |
| Flynn et al. | 1979 | USA | 60 | M | 1 | Alive | Low |
| Dresser et al. | 1979 | USA | 28 | M | 1 | Alive | High |
| Come et al. | 1979 | USA | 57 | F | 1 | Alive | High |
| Pernod et al. | 1978 | France | 29 | M | 1 | Alive | Low |
| Roguin et al. | 1977 | Israel | 22 | F | 1 | Alive | Low |
| Meyers et al. | 1977 | USA | 58 | M | 1 | Alive | Low |
| Gustafson et al. | 1977 | Sweden | 31 | M | 1 | Alive | Low |
| Fauvre et al. | 1977 | USA | 60 | M | 2 | Alive | Moderate |
|  |  |  | 57 | F |  | Alive |  |
| Burech et al. | 1977 | USA | 12 | F | 1 | Alive | Low |
| Yuste et al. | 1976 | Spain | 41 | M | 1 | Alive | Low |
| Symbas et al. | 1976 | USA | - | - | 1 | Alive | High |
|  |  |  | - | - | 1 | Alive |  |
|  |  |  | - | - | 1 | Alive |  |
| Siltanen et al. | 1976 | Finland | 33 | F | 2 | Died | Moderate |
|  |  |  | 16 | M |  | Alive |  |
| Muroff et al. | 1976 | USA | 61 | F | 1 | Alive | Low |
| Liebler et al. | 1976 | USA | 34 | M | 1 | Alive | Moderate |
| Fitterer et al. | 1976 | USA | 15 | F | 1 | Alive | Moderate |
| Berman et al. | 1976 | Canada | 53 | F | 1 | Alive | Moderate |
| Atsuchi et al. | 1976 | Japan | 22 | M | 1 | Alive | Moderate |
| Farah et al. | 1975 | USA | 21 | M | 1 | Alive | Moderate |
| Duvernoy et al. | 1975 | USA | 50 | F | 3 | Alive | Low |
|  |  |  | 54 | F |  | Alive |  |
|  |  |  | 7 | M |  | Died |  |
| Hartstein et al. | 1973 | USA | 49 | M | 1 | Alive | Low |
| Ferrans et al. | 1973 | USA | 46 | M | 2 | Alive | High |
|  |  |  | 51 | F |  | Alive |  |
| Waxler et al. | 1972 | USA | 40 | F | 1 | Alive | Low |
| Nasser et al. | 1972 | India | 58 | F | 2 | Alive | Low |
|  |  |  | 33 | F |  | Alive |  |
| Goldschlager et al. | 1972 | USA | 56 | F | 1 | Alive | Low |
| Croxson et al. | 1972 | UK | 17 | F | 1 | Alive | High |
| Collins et al. | 1972 | USA | 33 | M | 3 | Died (low cardiac output) | Moderate |
|  |  |  | 51 | F |  | Died (pneumococcal septicemia) |  |
|  |  |  | 66 | F |  | Alive |  |
| Chung et al. | 1971 | Australia | 53 | F | 1 | Alive | Low |
| Talley et al. | 1970 | USA | 49 | F | 1 | Alive | Low |
| Roberts et al. | 1970 | Canada | 38 | F | 1 | Alive | Moderate |
| Hattler Jr et al. | 1970 | UK | - | F | 3 | Alive: 3 (100%) | Moderate |
| Sievers et al. | 1970 | Sweden | 60 | F | 1 | Alive | Low |
| Martin et al. | 1969 | USA | 60 | F | 1 | Alive | Low |
| Marpole et al. | 1969 | USA | 73 | M | 1 | Alive | Low |
| Hansen et al. | 1969 | USA | 51 | F | 2 | Alive | Low |
|  |  |  | 53 | M |  | Alive |  |
| Miller et al. | 1968 | UK | 45 | F | 1 | Alive | Low |
| Matsushita et al. | 1968 | Japan | 37 | F | 1 | Died | Low |
| Fluck et al. | 1968 | UK | 41 | M | 1 | Alive | Low |
| Currey et al. | 1967 | UK | 38 | F | 1 | Alive | Low |
| sanyal et al. | 1967 | USA | 7 months | M | 1 | Alive | Low |
| Holswade et al. | 1966 | USA | 51 | F | 1 | Alive | Low |
| Krikler et al. | 1966 | England | 44 | M | 1 | Died | Low |
| Holswade et al. | 1965 | USA | 57 | F | 1 | Died | Moderate |
| Willman et al. | 1965 | USA | 61 | F | 1 | Alive | Moderate |
| Heath et al. | 1964 | UK | 35 | F | 1 | Died | Moderate |
| Morrissey et al. | 1963 | USA | 38 | F | 2 | Died | Low |
|  |  |  | 47 | F |  | Alive |  |
| Brandfonbrener et al. | 1963 | USA | 39 | M | 1 | Alive | Low |
| Sannerstedt et al. | 1962 | Sweden | 56 | F | 1 | Alive | Low |
| Emanuel et al. | 1962 | UK | 26 | F | 1 | Died | Low |
| Barlow et al. | 1962 | South Africa | 49 | F | 1 | Alive | Low |
| Cumming et al. | 1961 | Canada | 11 | M | 1 | Died | Low |
| Adams et al. | 1961 | USA | 33 | M | 1 | Died | Low |
| Ashman et al. | 1960 | USA | 38 | M | 1 | Alive | Low |
| Taber et al. | 1960 | USA | 51 | F | 2 | Alive | Low |
|  |  |  | 54 | F |  | Alive |  |
| Belle et al. | 1959 | USA | 43 | F | 1 | Died | Low |
| Cooley et al. | 1959 | USA | 43 | F | 2 | Died | Moderate |
|  |  |  | 48 | F |  | Alive |  |
| Capmpeau et al. | 1959 | Canada | 47 | M | 1 | Alive | Low |
| Lyons et al. | 1958 | USA | 51 | M | 1 | Died | Moderate |
| Krčílková et al. | 1958 | Czechoslovakia | 14 | F | 1 | Alive | Moderate |
| Coates et al. | 1958 | USA | 50 | F | 1 | Alive | Low |
| Ellis et al. | 1958 | USA | 48 | M | 1 | Alive | Low |
| Bahnson et al. | 1957 | USA | 54 | F | 1 | Died | Moderate |
| Paquet et al. | 1956 | Canada | 43 | F | 2 | Died | Moderate |
|  |  |  | 39 | M |  | Died |  |
| Buenger et al. | 1956 | USA | 16 | M | 1 | Died | Low |
| Macoun et al. | 1949 | UK | 64 | F | 1 | Died | Low |

**References**

1. Abdallah H, Michetti J, Ashour H, El Hendawy S, Al Khamees K, Demers P, et al. Fifteen-year experience in the surgical management of right atrial myxoma. 2017;

2. Abdelaziz A, Abdelgawad A, Ramadan MM, Osama M. A new complication of transesophageal echocardiography: pulmonary embolization of a right atrial myxoma. The Journal of Thoracic and Cardiovascular Surgery. 2015;149(5):e79–81.

3. Acikel S, Bozkaya O, Kiziltepe U. The diagnosis and clinical importance of tumour neovascularisation from coronary artery to right atrial myxoma. Kardiologia Polska (Polish Heart Journal). 2012;70(5):501–4.

4. Adams CW, Collins HA, Dummit ES, Allen JH. Intracardiac myxomas and thrombi: clinical manifestations, pathology and treatment. The American Journal of Cardiology. 1961;7(2):176–87.

5. Affronti A, Di Bella I, Prontera P, Da Col U, Ramoni E, Donti E, et al. Obstruction of the tricuspid valve orifice by a huge right atrial myxoma associated with the Carney complex: a case report. Journal of Cardiac Surgery. 2010;25(6):674–6.

6. Agostini D, Babatasi G, Galateau F, Grollier G, Potier JC, Bouvard G. Detection of cardiac myxoma by F-18 FDG PET. Clin Nucl Med. 1999;24(3):159–60.

7. Agrawal R, Sharma A, Nath RK, Pandit BN. Massive right atrial myxoma presenting as congestive heart failure: an unusual presentation of a rare tumour. BMJ Case Rep. 2018;2018.

8. Agstam S, Kumar B, Dahiya N, Guleria VS. Giant right atrial myxoma presenting as right heart failure: a rare manifestation. BMJ Case Reports CP. 2020;13(3):e230461.

9. Ahmed AK, Rajendran R, Shaukat N, Nishtar S, McAdam J. A rare case of atrial myxoma with biatrial extension. Int J Cardiol. 2008;127(2):e50-1.

10. Al Ani H. Adult Ebstein anomaly with right atrial myxoma. Iraqi postgraduate Medical Journal. 2006;5(2).

11. AL HUSSEIN H, AL HUSSEIN H, PASTORELLO Y, HARPA M, BĂLĂU R, GHIRAGOSIAN C, et al. GIANT RIGHT ATRIAL MYXOMA–CASE REPORT. Acta Medica Transilvanica. 2019;24(2):39–40.

12. Al Sergani R, Alamro B, Al Admawi M, Elmahi I, Iannuzzo G, Cittadini A, et al. Three dimensional echocardiographic imaging of multiple recurrent myxomas. Monaldi Archives for Chest Disease. 2020;90(1).

13. Alam M, Sun I, Smith S. Transesophageal echocardiographic evaluation of right atrial mass lesions. J Am Soc Echocardiogr. 1991;4(4):331–7.

14. Alamri Y, Lau YY, Lainchbury J. Large right atrial myxoma presenting with heart failure. ANZ Journal of Surgery. 2019;89(10):1341–2.

15. Alessio CF Mammana Liborio Francesco, De Donno Filomena Bruna RAS and P, Francesco. Right Atrial Myxoma in Patients with Metastatic Breast Cancer: Multidisciplinary Approach and Surgical Timing. Journal of Biomedical Research & Environmental Sciences. 2022;

16. Alizade E, Karabay CY, Kilicgedik A, Pala S, Kirma C. A giant right atrial myxoma demonstrated by RT-3D transesophageal echocardiography and magnetic resonance imaging. Cardiology Journal. 2011;18(3):320–1.

17. Alizadehasl A. Right Atrial Myxoma. Case-Based Clinical Cardiology. 2021;69–73.

18. Alsafwah S, Lababidi Z. Recurrent pulmonary embolism originating from right atrial myxoma. Journal of the American Society of Echocardiography. 2001;14(4):305–7.

19. Altujjar M, Zaiem F, Sheehan E, Gan W, Mhanna M, Khokher W, et al. Rare Case of 6 cm Right Atrial Myxoma in Patient with Synchronous Endometrial Adenocarcinoma. Case Rep Cardiol. 2021;2021:4657117–4657117.

20. Anagnostopoulos GK, Margantinis G, Kostopoulos P, Papadopoulou G, Roulias A, Sakorafas G, et al. Budd-Chiari syndrome and portal vein thrombosis due to right atrial myxoma. The Annals of thoracic surgery. 2004;78(1):333–4.

21. Ancona R, Comenale Pinto S, Caso P, Di Palma V, Pisacane F, Martiniello AR, et al. Right atrial mass following transcatheter radiofrequency ablation for recurrent atrial fibrillation: thrombus, endocarditis or mixoma? Monaldi Arch Chest Dis. 2009;72(1):40–2.

22. Animashaun I, Akinseye OA, Akinseye LI, Akinboboye OO. Right Atrial Myxoma and Syncope. Am J Case Rep. 2015;16:645–7.

23. Anitha K, Arulmozhi S. Anaesthetic Management of Right Atrial Myxoma-A Case report. University Journal of Medicine and Medical Specialities. 2021;7(4).

24. Aoyagi S, Tayama E, Yokokura Y, Yokokura H. Right atrial myxoma in a patient presenting with syncope. Kurume Med J. 2004;51(1):91–3.

25. Arnáiz-García ME, González-Santos JM, López-Rodríguez J, Dalmau-Sorlí MJ. Case images: giant right atrial myxoma mimicking a thrombus. Turk Kardiyol Dern Ars. 2014;42(1):113–113.

26. Ashman H, Zaroff LI, Baronofsky I. Right atrial myxoma. Diagnosis during life: successful surgical removal. Am J Med. 1960;28:487–96.

27. Atherton D, Pitcher D, Wells R, MacDonald D. A syndrome of various cutaneous pigmented lesions, myxoid neurofibromata and atrial myxoma: the NAME syndrome. British Journal of Dermatology. 1980;103(4):421–9.

28. Atipo-Galloye R, Sayeh R, Mitsomoy M, Loubna C. A rare giant right atrial myxoma arising from crista terminalis. Egyptian Heart Journal. 2013;65(4):329–32.

29. Atsuchi Y, Nagai Y, Nakamura K, Komatsu Y, Osamura Y. Echocardiographic diagnosis of prolapsing right atrial myxoma. Jpn Heart J. 1976;17(6):798–803.

30. Attili AK, Gebker R, Cascade PN. Radiological reasoning: right atrial mass. American Journal of Roentgenology. 2007;188(6_supplement):S26–30.

31. Ayan F, Koldas L, Karpuz H, Baslar Z, Sirmaci N. Asymptomatic giant prolapsing right atrial myxoma: Comparison of transthoracic and transesophageal echocardiography in pre-operative evaluation. Journal of Clinical and Basic Cardiology. 2000;3(3):197–8.

32. Aydın C, Taşal A, Ay Y, Vatankulu MA, İnan B, Bacaksız A. A giant right atrial villous myxoma with simultaneous pulmonary embolism. International Journal of Surgery Case Reports. 2013;5(4):206–8.

33. Azevedo O, Almeida J, Nolasco T, Medeiros R, Casanova J, Bartosch C, et al. Massive right atrial myxoma presenting as syncope and exertional dyspnea: case report. Cardiovascular Ultrasound. 2010;8:1–3.

34. Bahnson HT, Spencer FC, Andrus EC. Diagnosis and treatment of intracavitary myxomas of the heart. Annals of Surgery. 1957;145(6):915.

35. Bakri YN, Subhi J, Fawzi M. Right atrial myxoma presenting as post partum ascites and fever of unknown origin. Acta Obstet Gynecol Scand. 1992;71(1):74–5.

36. Bandyopadhyay S, Raychaudhuri T. Acute Reduction of Venous Return in a Patient With a Giant Right Atrial Myxoma. Anesthesia & Analgesia. 2017;125(1):52–5.

37. Banerjee A, Davenport A. Ascites due to right atrial myxoma in a haemodialysis patient. BMC Nephrol. 2006;7:4–4.

38. Barakizou H, Bayoudh F. Intermittent Prolapse of Right Atrial Myxoma in a Four-Year-Old Girl. Journal of Comprehensive Pediatrics. 2014;5(4).

39. Barlis P, Lim EJ, Gow PJ, Seevanayagam S, Calafiore P, Chan RK. Giant cardiac myxoma. Heart Lung Circ. 2007;16(5):389–91.

40. Barlow J, Fuller D, Denny M. A case of right atrial myxoma with special reference to an unusual. British Heart Journal. 1962;24:120–5.

41. Baronciani D, Angelucci E, Polchi P, Martinelli F, Mariotti E, Marzocchi A, et al. An unusual marrow transplant complication: cardiac myxoma. Bone marrow transplantation. 1997;21(8):825–7.

42. Basso C, Valente M, Casarotto D, Thiene G. Cardiac lithomyxoma. American Journal of Cardiology. 1997;80(9):1249–51.

43. Batinić T, Bulat C, Karabuva S, Zekanović D, Šušak Z, Bonacin D, et al. Background of extreme weight loss and weakness–right atrial myxoma. Medica Jadertina. 2013;43(1–2):73–6.

44. Battellini R, Bossert T, Areta M, Navia D. Successful surgical treatment of a right atrial myxoma complicated by pulmonary embolism. Interact Cardiovasc Thorac Surg. 2003;2(4):555–7.

45. Beiras-Fernandez A, Kornberger A, El-Beyrouti H, Vahl CF. Giant right atrial myxoma: A rare cause of cardiovascular collapse. SAGE Open Medical Case Reports. 2019;7:2050313X19841461.

46. Belle MS. Right atrial myxoma. Circulation. 1959;19(6):910–7.

47. Berger MD, Schneider J, Ballmer PE, Eckhardt BP, Dommann-Scherrer C. Mucin-producing adenocarcinoma arising in an atrial myxoma. Annals of diagnostic pathology. 2013;17(1):104–7.

48. Berman ND, McLaughlin PR, Bigelow WG, Morch JE. Angiographic demonstration of blood supply of right atrial myxoma. Br Heart J. 1976;38(7):764–6.

49. Bezgin T, Elveran A, Doan C, Karagöz A, Karabay C, Çanga Y, et al. Intermittent severe tricuspid stenosis caused by right atrial myxoma originating from the interatrial septum. Herz. 2014;39(3):412.

50. Bhalerao UD, Bhosle KN, Joshi MM, Siddiqui AT. Familial atrial myxoma in three generations: case report. INDIAN JOURNAL OF THORACIC AND CARDIOVASCULAR SURGERY. 2014;30(3):232–4.

51. Bharath V, Hote MP. Large Right Atrial Myxoma Masquerading as Malignancy. Journal of Cardiac Critical Care TSS. 2021;5(02):159–61.

52. Bhatti Z, Volodarskiy A. RIGHT ATRIAL MYXOMA IN PREGNANCY. Chest. 2019;156(4):A1799.

53. Bhosle SH, Niturkar GD, Rathod VM, Zine KU. Fatal pulmonary embolism due to asymptomatic right atrial myxoma: A case report. Journal of Indian Academy of Forensic Medicine. 2019;41(4):274–5.

54. Bilku RS, Loubani M, Been M, Patel RL. Massive right atrial myxoma causing exertional dyspnoea. Eur J Echocardiogr. 2008;9(1):130–2.

55. Birincioglu CL, Ulus AT, Katircioglu SF, Yamak B, Kale A, Aydog G, et al. Right atrial myxoma originating from tricuspid septal leaflet. Asian Cardiovascular and Thoracic Annals. 1999;7(1):59–61.

56. Bitner M, Jaszewski R, Wojtasik L, Zaslonka J. Unusual course of right atrial myxoma, masked by acute abdominal pain, and complicated by pulmonary embolus. Scandinavian Cardiovascular Journal. 1998;32(6):371–3.

57. Bortolotti U, Faggian G, Mazzucco A, Milano A, Thiene G, Fasoli G, et al. Right atrial myxoma originating from the inferior vena cava. Ann Thorac Surg. 1990;49(6):1000–2.

58. Bortolotti U, Maraglino G, Rubino M, Santini F, Mazzucco A, Milano A, et al. Surgical excision of intracardiac myxomas: a 20-year follow-up. Ann Thorac Surg. 1990;49(3):449–53.

59. Boutayeb A, Marmade L, Bensouda A, Moughil S. Right atrial myxoma and severe left ventricular dysfunction: which explanations? Which management? Heart, Lung and Circulation. 2012;22(4):309–11.

60. Brandfonbrener M, Kroll G, Borden CW, Lewis FJ. Right atrial myxoma successfully removed. Arch Intern Med. 1963;111:814–8.

61. Buenger RE, Paul O, Fell EH. Calcified polyp of the heart. Radiology. 1956;67(4):531–6.

62. Bulić P, Benko K, Hlača Caput T, Lulić D, Matijević Rončević S, Matulić S, et al. Giant right atrial myxoma and ovarian formation: a case report. Cardiologia Croatica. 2021;16(5–6):206–206.

63. Burech DL, Teske DW, Haynes RE. Right atrial myxoma in a child. American Journal of Diseases of Children. 1977;131(7):750–2.

64. Burke AP, Virmani R. Cardiac myxoma: a clinicopathologic study. American journal of clinical pathology. 1993;100(6):671–80.

65. Burns ER, Schulman IC, Murphy Jr MJ. Hematologic manifestations and etiology of atrial myxoma. AMERICAN JOURNAL OF THE MEDICAL SCIENCES. 1982;284(2):17–22.

66. Butler MJ, Adams Jr HP, Hiratzka LF. Recurrent cerebral embolism from a right atrial myxoma. Ann Neurol. 1986;19(6):608–9.

67. Çakmak Gökçe BM, Arslan G, Haberal C. The management of fast-track cardiac anesthesia in a patient with right atrial myxoma. Turkish Journal of Thoracic and Cardiovascular Surgery. 2012;20(3):625–8.

68. Campeau L, David P. Myxoma of the heart. Canadian Medical Association Journal. 1960;82(11):586.

69. Canale LS, Colafranceschi AS, Leal Botelho ES, de Oliveira Monteiro AJ. Surgical treatment of right atrial myxoma complicated with pulmonary embolism. Interact Cardiovasc Thorac Surg. 2009;9(3):535–6.

70. Cappell MS, Lapin S, Rose M. Large right atrial myxoma containing gastric heterotopia presenting with dyspnea and bilateral leg edema due to pulmonary emboli and cardiovascular obstruction: the first known report of gastric heterotopia in the cardiovascular system. Digestive diseases and sciences. 2008;53:405–9.

71. Carrasco CA, Rojas-Salazar D, Chiorino R, Venega JC, Wohllk N. Melanotic nonpsammomatous trigeminal schwannoma as the first manifestation of Carney complex: case report. Neurosurgery. 2006;59(6):E1334-5; discussion E1335.

72. Carvalho MS, Andrade MJ, Abecasis J, Gouveia R, Branco L, Neves JP, et al. Understanding cardiac myxoma recurrence: A case report. Revista Portuguesa de Cardiologia. 2013;32(3):239–42.

73. Cavero MA, Cristóbal C, González M, Gallego JC, Oteo JF, Artaza M. Fatal pulmonary embolization of a right atrial mass during transesophageal echocardiography. Journal of the American Society of Echocardiography. 1998;11(4):397–8.

74. CERVETTI MR, PICCININI F, MUÑOZ Á. Right Atrial Myxoma Resection and Atrial Wall Reconstruction. Revista Argentina de Cardiología. 2018;86(3):214.

75. Chamsi-Pasha MA, Anwar AM, Nosir YF, Abukhudair W, Ashmeg A, Chamsi-Pasha H. Right atrial myxoma associated with an atrial septal defect by real-time three-dimensional echocardiography. Eur J Echocardiogr. 2009;10(2):362–4.

76. Changal K, Nawaz H. ASYMPTOMATIC PATIENT WITH COMPLETE EMBOLIZATION OF RIGHT ATRIAL MYXOMA CAUSING A LARGE PULMONARY EMBOLISM. Journal of the American College of Cardiology. 2018;71(11S):A2453–A2453.

77. Charokopos NA, Rouska E, Pliakos C, Pagourelias ED, Artemiou P, Foroulis C, et al. Atypical atrial myxomas in two asymptomatic patients: a case report. Cardiovascular ultrasound. 2009;7:1–3.

78. Cheema U, Thomas J. A giant right atrial myxoma presenting as acute pulmonary emboli. European Heart Journal–Cardiovascular Imaging. 2012;13(9):799–799.

79. Chen R, Deng X, Luo J, Huang P. Calcified inferior vena cava and right atrial myxoma in an 18-month-old male: a case report. Medicine. 2018;97(25).

80. Chhabra A, McClung JA, Kalapatapu S, Lafaro RJ, Fallon 3rd JT, Aronow WS. Giant myxoma causing heart failure symptoms. Am J Case Rep. 2012;13:29–32.

81. Choi J, de Costa A, Sabetai MM. Surgical management of a giant right atrial myxoma. Journal of Surgical Case Reports. 2018;2018(10):rjy288.

82. Chu SH, Andrews D, Watanabe Y. Emergency surgical embolectomy for pulmonary emboli after failed thrombolysis. Asian Cardiovascular and Thoracic Annals. 2009;17(3):297–9.

83. Chung EK, Dean HM. Right atrial myxoma. Unusual post-operative complications. Jpn Heart J. 1971;12(3):316–23.

84. Coates Jr EO, Drake EH. Myxoma of the right atrium, with variable right-to-left shunt: clinical and physiologic observations and report of a case with successful operative removal. New England Journal of Medicine. 1958;259(4):165–9.

85. Collins HA, Collins IS. Clinical experience with cardiac myxoma. Ann Thorac Surg. 1972;13(5):450–7.

86. Come PC, Kurland GS, Vine HS. Two dimensional echocardiography in differentiating right atrial and tricuspid valve mass lesions. Am J Cardiol. 1979;44(6):1207–12.

87. Contreras CAS, Arias MRG, Navarrete MA, Silva SO, Méndez H, Frausto AA, et al. GIANT RIGHT ATRIAL MYXOMA, UNUSUAL FEATURES. Journal of the American College of Cardiology. 2021;77(18_Supplement_1):2680–2680.

88. COOLEY EA, Morris GC, ATTAR S. Cardiac myxoma: surgical treatment in four cases. AMA Archives of Surgery. 1959;78(3):410–7.

89. Coughlin WF, Knott PE. Right atrial myxoma. A cause of septic pulmonary emboli in an adolescent female. J Adolesc Health Care. 1990;11(4):351–4.

90. Croxson RS, Jewitt D, Bentall HH, Cleland WP, Kristinsson A, Goodwin JF. Long-term follow-up of atrial myxoma. Br Heart J. 1972;34(10):1018–23.

91. Cujec B, Ulmer B, McKaigney JP, Bharadwaj B. Right atrial myxoma presenting as Budd-Chiari syndrome. Ann Thorac Surg. 1987;44(6):658–9.

92. Cumming G, Finkel K. Intracardiac myxoma involving the right and left atria in a young patient. The Journal of Pediatrics. 1961;58(4):559–67.

93. Currey HL, Mathews JA, Robinson J. Right atrial myxoma mimicking a rheumatic disorder. Br Med J. 1967;1(5539):547–8.

94. Darwazah AK, Eida M, Batrawy M. Myxoma at junction of inferior vena cava and right atrium: surgical excision. Texas Heart Institute Journal. 2011;38(5):591.

95. De Carli S, Sechi LA, Ciani R, Barillari G, Dolcetti G, Bartoli E. Right atrial myxoma with pulmonary embolism. Cardiology. 1994;84(4–5):368–72.

96. De Peppo AP, Sommariva L, Pellegrino A, Persiani M, Chiariello L. Cardiac myxoma arising from the inferior vena cava. Texas Heart Institute Journal. 1992;19(4):288.

97. de Roos A, Weijers E, van Duinen S, van der Wall EE. Calcified right atrial myxoma demonstrated by magnetic resonance imaging. Chest. 1989;95(2):478–9.

98. Demirtürk OS, Gülcan Ö, Tünel HA, Güvener M. De novo right atrial myxoma detected nine months after atrial septal defect closure. Türk Göğüs Kalp Damar Cerrahisi Dergisi. 2012;20:926–8.

99. Depelchin P, Ibrahim T, Sarot J, Thirion M. Detection of right atrial myxoma by Fourier phase analysis. Eur J Nucl Med. 1987;13(6):318–20.

100. Deshpande A, Kumar S, Chopra P. Recurrent, biatrial, familial cardiac myxomas. International journal of cardiology. 1994;47(1):71–3.

101. Devig CM, Clark CTA, Aaron CB. Cardiac myxoma arising from the inferior vena cava. Chest. 1980;78(5):784–6.

102. Dewi ISK, Winaya IA. Right atrial tumor suspected myxoma in an 11-year-old girl. Paediatrica Indonesiana. 2008;48(1):54–8.

103. Dey B, Raphael V, Khonglah Y, Jyoti PK. Cardiac myxoma with glandular elements: An unusual histologic variant with a brief review of the literature. Journal of the Practice of Cardiovascular Sciences. 2019;5(2):119–21.

104. Dharmalingam SK, Sahajanandan R. Intraoperative transesophageal echocardiography assessment of right atrial myxoma resulting in a change of the surgical plan. Ann Card Anaesth. 2014;17(4):306–8.

105. Dianzumba SB, Char G. Large calcified right atrial myxoma in a newborn. Rare cause of neonatal death. British heart journal. 1982;48(2):177.

106. Dindaş F, Cerik İB, Koyun E, Turgut OO. A Rare Clinical Presentation Caused by Atrial Myxoma: Right Heart Failure. Archives of Current Medical Research. 2020;2(1):65–8.

107. Disney L, Gunn T, Klimkina O, Keshavamurthy S. Giant right atrial myxoma associated with thrombocytopaenia. Heart, Lung and Circulation. 2021;30(12):e127–8.

108. Dobson R. Diagnosis of right atrial myxoma by cardiac MRI: an unexpected consequence of clinical research. Heart. 2012;98(20):1540–1540.

109. Dong A, Lu J, Zuo C. Multiple Peripheral Pulmonary Artery Aneurysms in Association With a Right Atrial Myxoma. Circulation. 2016;133(4):444–6.

110. Dorobantu LF, Chioncel O, Pasare A, Usurelu DL, Bubenek-Turconi IS, Iliescu VA. An unusual association: right atrial myxoma and severe left ventricular dysfunction. Case report and review of the literature. In 2014. p. 420.

111. Dresser TP, Rao BR, Winebright JW. Nuclear angiocardiogram to demonstrate right atrial myxoma. Clin Nucl Med. 1979;4(5):206–7.

112. Durand P, Furudoi A, Parrens M, Lazaro E, Viallard J, Rivière E. Complete remission of immune thrombocytopenia in a 30‐year old woman after excision of a right atrial myxoma. British Journal of Haematology. 2019;187(1):e18–9.

113. Duvernoy WF, Drake EH, Reddy MS, Karo JJ. Atrial myxoma: a review of 9 cases. Cardiology. 1975;60(4):206–19.

114. Eckhardt BP, Dommann-Scherrer C, Stuckmann G, Zollikofer CL, Wentz KU. Giant cardiac myxoma with malignant transfromed glandular structures. European Radiology. 2003;13(9):2099–102.

115. Eftekharzadeh P, Ahmed S. Acute Coronary Syndrome or Right Atrial Cardiac Myxoma? An Atypical Presentation. Cureus. 2021;13(10).

116. Elhamamsy M, Aldemerdash A, Zahran F, Bakir B, Alanazi N, Awadallah Y, et al. Persistent left superior vena cava in patient with right atrial myxoma undergoing open heart surgery. A case report and review of literature. SAUDI JOURNAL OF ANAESTHESIA. 2020;14(2):217–20.

117. Ellis Jr FH, Mankin HT, Burchell HB. Myxoma of the atrium: successful surgical treatment in two cases. Medical Clinics of North America. 1958;42(4):1087–99.

118. Elmusa K, Haddad R, Arnaout MS. Atypical and unusual atrial myxoma. J Med Liban. 2004;52(1):48–50.

119. Emanuel RW, Lloyd WE. RIGHT ATRIAL MYXOMA MISTAKEN FOR CONSTRICTIVE PERICARDITIS. Br Heart J. 1962;24(6):796–800.

120. Essandoh M, Andritsos M, Kilic A, Crestanello J. Anesthetic Management of a Patient With a Giant Right Atrial Myxoma. Semin Cardiothorac Vasc Anesth. 2016;20(1):104–9.

121. Fabijanić D, Rudeš I, Kardum D, Radić M, Glavaš D, Lozo P. Pulmonary embolism due to the right atrial myxoma. Collegium antropologicum. 2006;30(4):933–6.

122. Fácila-Rubio L, Nuñez-Villota JE, Losada-Casares A, Otero-Coto E, Marin-Pardo J, Ferreres-Franco J, et al. Hemoptysis as an unusual manifestation of right atrial myxoma. INTERNATIONAL JOURNAL OF CARDIOLOGY. 2002;86(1):119–21.

123. Fagan Jr LF, Castello R, Barner H, Moran M, Labovitz AJ. Transesophageal echocardiographic diagnosis of recurrent right ventricular myxoma 2 years after excision of right atrial myxoma. Am Heart J. 1990;120(6 Pt 1):1456–8.

124. Faisal AWK, Riaz W, Naveed T, Iqbal MH, Hassan A, Ali SA. Tricuspid valve myxoma: An uncommon presentation of a common tumour. Echocardiography. 2020;

125. Fang YM, Dean R, Figueroa R. Right atrial myxoma mimicking an atrial thrombus in the third trimester of pregnancy. J Matern Fetal Neonatal Med. 2007;20(1):77–8.

126. Farah MG. Familial atrial myxoma. Ann Intern Med. 1975;83(3):358–60.

127. Faruqui AM, SYED SAl, Shareef M. RIGHT ATRIAL MYXOMA. Pakistan Heart Journal. 1980;13(3).

128. Fatimi SH, Deedar-Ali-Khawaja R, Kiani SK. Imaging and intervention of paraneoplastic effect of a right atrial myxoma on factor VII activity levels. The Annals of thoracic surgery. 2011;91(1):278–81.

129. Fauvre F, Johns Jr VJ, Jutzy RV. Cardiac myxomas: report of eight cases with successful excision. West J Med. 1977;127(4):284–91.

130. Fayad G, Modine T, Letourneau T, Hervieux E, Veraeghe R, Bekhti H, et al. Paradoxical cerebral embolism and right atrial myxoma. Journal of Cardiac Surgery. 2008;23(6):786–7.

131. Fernandes GC, Alejandro Pajares W, Amboss N, Salerno TA, Mendoza C. Right atrial myxoma with peripheral eosinophilia: Eosinophilia in cardiac myxoma. J Card Surg. 2020;35(2):507–10.

132. Ferrans VJ, Roberts WC. Structural features of cardiac myxomas. Histology, histochemistry, and electron microscopy. Human Pathology. 1973;4(1):111–46.

133. Filho JG, Sales DL de S, Borges AEPP, Leitão MC. Right atrial myxoma prolapsing into the right ventricle. Brazilian Journal of Cardiovascular Surgery. 2006;21:217–20.

134. Fitterer JD, Soicer MJ, Nelson CW. Echocardiographic demonstration of bilateral atrial myxomas. Chest. 1976;70(2):282–4.

135. Flint N, Siegel RJ, Bannykh S, Luthringer DJ. Bi-atrial cardiac myxoma with glandular differentiation: a case report with detailed radiologic-pathologic correlation. European Heart Journal-Case Reports. 2018;2(2):yty045.

136. Fluck DC, Lopez-Bescos L, Baker CG. Calcified Right Atrial Myxoma Producing Tricuspid Incompetence. Journal of the Royal Society of Medicine. 1968;61:1115–8.

137. Flynn W, Garcia-Rinaldi R, Roehm Jr JO, Crawford ES. Surgical treatment of infected right atrial myxoma. Ann Thorac Surg. 1979;27(3):242–5.

138. Forbes LM, Hensley ND, Miller YE. A 58-year-old woman with a history of cardiac myxoma presents with pulmonary nodules. Chest. 2021;160(4):e351–5.

139. Fox JJ, Bonavita GJ, Sethi V. Right atrial myxoma associated with vascular malformation and atypical chest pain. Cathet Cardiovasc Diagn. 1992;26(2):127–9.

140. Fracasso T, Varchmin-Schultheiss K. Sudden death due to pulmonary embolism from right atrial myxoma. International journal of legal medicine. 2009;123(2):157–9.

141. Frishman W, Factor S, Jordan A, Hellman C, Elkayam U, LeJemtel T, et al. Right atrial myxoma: unusual clinical presentation and atypical glandular histology. Circulation. 1979;59(5):1070–5.

142. Fujiwara M, Watanabe H, Iino T, Koizumi M, Ito H. A Huge Right Atrial Myxoma Growing into Right Ventricule. Journal of Cardiac Failure. 2010;16(9):S165.

143. Funk M, Santana O, Lamelas J. Recurrent asymptomatic right atrial myxoma. Reviews in Cardiovascular Medicine. 2010;11(3):e176-80.

144. Fyke III FE, Seward JB, Edwards WD, Miller Jr FA, Reeder GS, Schattenberg TT, et al. Primary cardiac tumors: experience with 30 consecutive patients since the introduction of two-dimensional echocardiography. Journal of the American College of Cardiology. 1985;5(6):1465–73.

145. Garcia JD, Rodriguez Henriquez PJ, Balderas Juarez J, González Sánchez HR, González Gallo O, Palacios Herrera A. Hughes Stovin Syndrome as a Variant of Behçet Disease Associated With a Right Atrial Myxoma. J Clin Rheumatol. 2021;27(8s):S434-s436.

146. Garg A, Agrawal D, Sharma G. Right atrial myxoma with coexistent coronary artery disease - A rare combination. Journal of Cardiovascular Echography. 2020;30(2):100–3.

147. Gewehr DM, Neiverth A, Cavalcanti MS, Maestri TC, Haurani S, Kubrusly FB, et al. Fast growth rate of a right atrial myxoma. Einstein (Sao Paulo). 2022;20:eRC6478.

148. Ghasemi R, Motlagh FG, Nazari S, Yaghubi M. Huge mass in right side of the heart: A rare case report. ARYA ATHEROSCLEROSIS. 2016;12(6):291–4.

149. Giacalone A, Surlani A, Monga G. Simulation of right atrial cardiac myxoma by silent hepatocellular carcinoma. Pathology-Research and Practice. 1996;192(12):1270–3.

150. Gilkey SH. Right atrial myxoma. Semin Roentgenol. 1987;22(2):85–6.

151. Gladden JR, Dreiling RJ, Gollub SB, Bixler Ii TJ, Dunn MI. Two-dimensional echocardiographic features of multiple right atrial myxomas. The American Journal of Cardiology. 1983;52(10):1364–5.

152. Gogas BD, Rallidis LS, Iliodromitis EK, Kremastinos DT. Right atrial myxoma presenting as syncope due to pulmonary embolism in a previously asymptomatic patient. International Journal of Cardiology. 2011;148(2):e34–6.

153. Göksel S, Kural T. Lentiginosis and right atrial myxoma. Eur Heart J. 1989;10(8):769–71.

154. Goksuluk H, Doğan O, Dokumacı B, Ozer I. An Atypical Giant Right Atrial Myxoma Presented with Minimal Symptoms. Int J Vasc Surg Med 2 (1): 015-017 DOI: 1017352/2455. 2016;5452.

155. Goldberg B, Romano A, Amato J, Valderrama E, Bierman F. Unique association of a rapidly growing right atrial myxoma in a child with double-outlet right ventricle. Pediatric cardiology. 1997;18:303–5.

156. Goldschlager A, Popper R, Goldschlager N, Gerbode F, Prozan G. Right atrial myxoma with right to left shunt and polycythemia presenting as congenital heart disease. Am J Cardiol. 1972;30(1):82–6.

157. Gomase S, Kuthe S, Sonkusale M. Case Report-Right atrial myxoma with total anomalous pulmonary venous connection in neonate. Int J Surg Case Rep. 2022;97:107438–107438.

158. Gong X, Yuan B, Yuan Y. Congenital hypofibrinogenemia with recurrent thromboembolism: A clinical case report. Thrombosis Update [Internet]. 2022;6. Available from: https://www.scopus.com/inward/record.uri?eid=2-s2.0-85128706807&doi=10.1016%2fj.tru.2022.100099&partnerID=40&md5=2584a454018ceb92ab3c0f50168af240

159. Gopalakrishnan M, Benjamin M, Kamath M, ThekkePurackal R. Cardiac myxoma mimicking extension of renal cell carcinoma. Interactive cardiovascular and thoracic surgery. 2010;10(5):816–7.

160. Göz M. Right atrial myxoma in a patient presenting with syncope. Turkish Journal of Thoracic and Cardiovascular Surgery. 2006;14(4).

161. Grebenc ML, Rosado-de-Christenson ML, Green CE, Burke AP, Galvin JR. Cardiac myxoma: imaging features in 83 patients. Radiographics. 2002;22(3):673–89.

162. Gromadziński L, Zelazny P, Zechowicz M, Moczulska B, Zwiernik B, Gimeła M, et al. Diffuse cerebral embolism as the first manifestation of the right atrial myxoma. Polish Annals of Medicine. 2015;22(2):136–8.

163. Grover A, Wahi PL. Right atrial myxoma. Chest. 1991;100(1):287–287.

164. Gu S, Liu Y, Yan J, Zhang X, Gao J, Xin Y, et al. Surgical treatment of right atrial myxoma and pulmonary embolism. Chin Med J (Engl). 2014;127(1):187–8.

165. Guhathakurta S, Riordan JP. Surgical treatment of right atrial myxoma. Texas Heart Institute Journal. 2000;27(1):61.

166. Guo H, Xu J, Xiong H, Hu S. Case studies of two related Chinese patients with Carney complex presenting with extensive cardiac myxomas and PRKAR1A gene mutation of c.491_492delTG. World Journal of Surgical Oncology [Internet]. 2015;13(1). Available from: https://www.scopus.com/inward/record.uri?eid=2-s2.0-84928741129&doi=10.1186%2fs12957-015-0470-4&partnerID=40&md5=697a556d0efbc9f51f71b11d67fd7acf

167. Gupta D, Molina E, Palma J, Macha M. Massive right atrial myxoma presenting with syncope. Heart and Vessels. 2006;21:256–7.

168. Gustafson AG, Edler IG, Dahlbäck OK. Bilateral Atrial Myxomas Diagnosed by Echocardiography. Acta Medica Scandinavica. 1977;201(1–6):391–4.

169. Haime M. Right atrial myxoma. J Card Surg. 20090929th ed. 2009 Nov;24(6):691–2.

170. Hamid MFA. A young woman with multiple intra cardiac mass, systemic symptoms and stroke. Eastern Journal of Medicine. 2017;22(4):214–7.

171. Han H, Li Y, Guo S, Yu X. Right atrial myxoma-induced syncope. Postgrad Med J. 2011;87(1028):438–9.

172. Hansen JF, Lyngborg K, Andersen M, Wennevold A. Right atrial myxoma. Acta Med Scand. 1969;186(3):165–71.

173. Hartstein ML, Wisoff BG, Meilman E, Hamby RI. Right atrial myxoma and arteriosclerotic heart disease. J Thorac Cardiovasc Surg. 1973;66(6):965–8.

174. Haruki N, Onohara T, Tsujimoto D, Iitsuka K, Kinugasa Y, Kato M, et al. Rapid-Growing Right Atrial Myxoma 7 Months After Catheter Ablation Under Anticoagulation Therapy - Serial Echocardiography and Computed Tomography. Circ J. 2018;82(10):2682–3.

175. Hattler Jr BG, Fuchs JC, Cosson R, Sabiston Jr DC. Atrial myxoma. An evaluation of clinical and laboratory manifestations. Ann Thorac Surg. 1970;10(1):65–74.

176. Hatz D, Danielson D, Nilas Young J, Amsterdam E. The impact of preoperative imaging for atypical atrial myxoma locations. Clin Cardiol. 2012;35(8):490–3.

177. Hayek ER, Haas AJ, Kamienski RW. Massive right atrial myxoma presenting as a chronic anemia. J Am Soc Echocardiogr. 2007;20(6):771.e1-2.

178. He J, Xia J, Ma X. A case of bilateral atrial myxoma straddling the foramen ovale. European Heart Journal. 2022;43(41):4440–4440.

179. He K, Bian L, Liang W, Wu Z. Fire at the gate ruins fish: pulmonary embolism caused by right atrial myxoma. Cardiovasc J Afr. 2022;33:1–3.

180. Heath D, Mackinnon J. Pulmonary hypertension due to myxoma of the right atrium. American Heart Journal. 1964;68(2):227–35.

181. Heck Jr HA, Gross CM, Houghton JL. Long-term severe pulmonary hypertension associated with right atrial myxoma. Chest. 1992;102(1):301–3.

182. Heidari A, Nourizadeh M, Najafi MH, Nourizadeh S, Assadinia N. Right atrial giant myxoma occupying the right ventricular cavity. Case Reports in Clinical Medicine. 2018;7(8):476–81.

183. Hemachandran M, Kakkar N, Khandelwal N. Giant-cell–rich myxoma of right atrium: An ultrastructural analysis. Cardiovascular Pathology. 2003;12(5):287–9.

184. Hendricksen DK, Gossman W. Atrial myxoma presenting as a pulmonary embolus. The American journal of emergency medicine. 1996;14(4):441–441.

185. Henmi S, Salica A, Scaffa R, D’Aleo S, Wolf LG, De Paulis R. Excision of large right atrial myxoma through a right mini thoracotomy. Journal of Surgical Case Reports. 2022;2022(5):rjac182.

186. Hinić S, Šarić J, Milojević P, Gavrilović J, Durmić T, Ninković N, et al. Benign tumors of the heart: Myxoma of the right atrium – a case report. Vojnosanitetski Pregled. 2018;75(5):512–5.

187. Holswade GR. RIGHT ATRIAL MYXOMA COMPLICATED BY A THROMBUS. Jama. 1965;193:306–8.

188. Holswade GR, Nydick I, Steinberg I. Successful removal of right atrial myxoma mistaken for liver and pericardial metastases. J Thorac Cardiovasc Surg. 1966;52(2):240–4.

189. Horne D, Jassal DS, Mysore S, Kirkpatrick ID, Freed DH, Hussain F. Multimodality imaging of a right atrial myxoma with pulmonary embolization. Can J Cardiol. 2012;28(4):516.e13-4.

190. Hsi DH, Sosa A, Miller W, Oren T, Koulova A, Coady MA. A giant right atrial myxoma—The growth rate and multi-modality imaging. Echocardiography. 2021;38(6):1057–60.

191. Huang LT, Tsai YS, Kan CD. Teaching NeuroImages: CRAO and silent brain infarcts caused by cardiac myxomas in Carney complex. Neurology. 2019;92(3):E286–7.

192. Hwang JJ, Lien WP, Kuan P, Hung CR, How SW. Atypical myxoma. Chest. 1991;100(2):550–1.

193. Iacobellis G, Di Gioia CR, Tamburrano G. Images in Cardiology: Asymptomatic right atrial myxoma in acromegalic man: a case of Carney complex. Heart. 2001;85(1):86–86.

194. Ibrahim M, Iliescu C, Safi HJ, Buja ML, McPherson DD, Fuentes F. Biatrial myxoma and cerebral ischemia successfully treated with intravenous thrombolytic therapy and surgical resection. Texas Heart Institute Journal. 2008;35(2):193.

195. Idir M, Oysel N, Guibaud JP, Labouyrie E, Roudaut R. Fragmentation of a right atrial myxoma presenting as a pulmonary embolism. Journal of the American Society of Echocardiography. 2000;13(1):61–3.

196. Ikeda A, Tsukada T, Konishi T, Matsuzaki K, Jikuya T, Hiramatsu Y. Right atrial myxoma with a large tumor embolus in the left pulmonary artery. J Surg Case Rep. 2014;2014(10).

197. Imai Y, Taketani T, Maemura K, Takeda N, Harada T, Nojiri T, et al. Genetic analysis in a patient with recurrent cardiac myxoma and endocrinopathy. Circulation Journal. 2005;69(8):994–5.

198. Jamidar HA, Webb SW, Adgey AA. Survival of a patient with an infected right atrial myxoma following surgery. Eur Heart J. 1988;9(1):110–3.

199. Jara-Palomares L, Serrano-Gotarredona MP, Lopez-Haldón J, Rodriguez-Puras MJ, Bibiloni-Lage I, Ruiz-Solano E, et al. Right atrium mass in a 28-year-old patient with pulmonary embolism taking contraceptives. Journal of Atherosclerosis and Thrombosis. 2011;18(9):829–32.

200. Jardine D, Lamont D. Right atrial myxoma mistaken for recurrent pulmonary thromboembolism. Heart. 1997;78(5):512–4.

201. Javeed M, Gruhonjic H, Kirkman T, Pitarys C, Akel R. A unique case of a right atrial myxoma infected with Escherichia coli. Cureus. 2022;14(5).

202. Jayaprasad N, Roy ST, Sajeev CG, Venugopal K. Right atrial myxoma. J Assoc Physicians India. 2006;54:125–125.

203. Jayaweera J, Kothalawala M, Sooriyar S. Infected tricuspid valve myxoma with Kodamaea ohmeri: case report. Indian Journal of Medical Microbiology. 2021;39(2):252–5.

204. Jia H, Xing Y, Zhang S, Wang Y. Hemodynamic management of a patient with a huge right atrium myxoma during thoracic vertebral surgery: A case report. Medicine. 2018;97(39).

205. Jovanova S, Lj GI, Kamcevska-Dobrkovic L. UNUSUALLY LARGE RIGHT ATRIAL MYXOMA PRESENTING WITH ATYPICAL CLINICAL MANIFESTATION. 2014;

206. Jung J, Hong YS, Lee CJ, Lim SH, Choi H, Lee S. Successful surgical treatment of a right atrial myxoma complicated by pulmonary embolism. Korean J Thorac Cardiovasc Surg. 2013;46(1):63–7.

207. Kale SB, Badkhal A, Kumar NM, Raghavan J. Right atrial mass after open heart surgery: tumour or thrombus? Heart Lung Circ. 2012;21(5):287–8.

208. Kaplan LJ, Weiman DS, VanDecker W, Sokil AB, Whitman GJ. Infected biatrial myxoma: Transesophageal echocardiography-guided surgical resection. The Annals of thoracic surgery. 1994;57(2):487–8.

209. Kapusta A, Lipiec P, Chrzanowski L, Foryś J, Kasprzak JD. [Untypical cause of heart failure--right atrial myxoma]. Pol Arch Med Wewn. 2007 Oct;117(10):470–2.

210. Karayannis G, Spanos P, Triposkiadis F, Skoularigis J. Right atrial myxoma involving the tricuspid annulus. Journal of Cardiac Surgery. 2010;25(5):533–533.

211. Karlof E, Salzberg SP, Anyanwu AC, Steinbock B, Filsoufi F. How fast does an atrial myxoma grow? The Annals of thoracic surgery. 2006;82(4):1510–2.

212. Kaya Ö, Ermiş H, Türkkan S, Aytemur ZA, Baysal T, Açıkgöz N. A right atrial myxoma mimicking pulmonary embolism: a case report. 2014;

213. Keenan DJ, Morton P, O’Kane HO. Right atrial myxoma and pulmonary embolism. Rational basis for investigation and treatment. Br Heart J. 1982;48(5):510–2.

214. Kejriwal NK, Tan J, Ullal RR, Alvarez JM. Atrial myxoma with coexistent coronary artery disease: a report of two cases. Heart Lung Circ. 2003;12(2):108–11.

215. Keller H, Stegaru B, Buss J, Genth K, Heene D. Pulmonary tumor embolism and right atrial myxoma detected by two-dimensional echocardiography. Am Heart J. 1985;110(4):881–4.

216. Keren A, Chenzbruna A, Schuger L, Milgarter E, Tzivoni D. The etiology of tumor plop in a patient with huge right atrial myxoma. Chest. 1989;95(5):1147–9.

217. Khansari N, Abdolhosseini M, Karimian K, Fariba F. Atypical Symptom and Clinical Features of Right Atrial Myxoma: A Case Report. 2022;

218. Knepper LE, Biller J, Adams Jr HP, Bruno A. Neurologic manifestations of atrial myxoma. A 12-year experience and review. Stroke. 1988;19(11):1435–40.

219. Kollias VD, Theodoropoulos SP, Yacoub MH. Right atrial appendage myxoma following recent coronary artery bypass grafting. Interactive Cardiovascular and Thoracic Surgery. 2004;3(1):195–7.

220. Komsuoǧlu B, Duman E, Komsuoǧlu SŞ. Familial atrial myxomas. INTERNATIONAL JOURNAL OF CARDIOLOGY. 1987;16(3):307–11.

221. Konecny T, Reeder G, Noseworthy PA, Konecny D, Carney JA, Asirvatham SJ. Percutaneous ablation and retrieval of a right atrial myxoma. Heart, Lung and Circulation. 2014;23(11):e244–7.

222. Kontogiorgi M, Kalodimou VE, Samanidis G, Vartela V, Tasouli A, Ghiatas A, et al. Recurrent cardiac myxoma in a 25 year old male: a DNA study. World Journal of Surgical Oncology. 2013;11:1–5.

223. Korkmaz AA, Tamtekin B, Onan B, Demir AS, Guden M, Uckurt Y. Combination of right atrial and left ventricular myxoma. The Annals of thoracic surgery. 2010;89(5):e33–5.

224. Krčílková M, Musil J, Navratil J, Olejnik O. The successful removal of a tumour from the right atrium under hypothermia. Thorax. 1958;13(3):173.

225. Krikler D, Friedman M. Right atrial myxoma. South African Medical Journal. 1966;40(47):1138–41.

226. Kumar SP, Kaul S, Saha PK, Miller MJ. Images in cardiology. Angiographic appearance of “tumour blush” produced by a large right atrial myxoma. Heart. 2006;92(6):751–751.

227. Kumar S, Khangarot S, Minhas HS, Satsangi DK. Multifocal Right Atrial Myxoma with Multiple Pulmonary Embolism. Journal of Cardiac Surgery: Including Mechanical and Biological Support for the Heart and Lungs. 2013;28(6):714–6.

228. Kumar T, Rajvanshi S, Sharma AK, Pandit N. “Double ball valve mechanism obstructing both right ventricular inflow and outflow”: Atypical presentation of right atrial myxoma presenting as right ventricular mass. Heart Views: The Official Journal of the Gulf Heart Association. 2017;18(4):141.

229. Kumary VS, Madhavan S, Akhil PC, Jayaprakash K, George R. Two-time recurrence of a right atrial myxoma. Proc (Bayl Univ Med Cent). 2015;28(4):507–8.

230. Kumpare MB, RaljeviÊ D, Milas K, DodiÊ D, JukiÊ N, PerπiÊ V. Right atrial myxoma associated with atrial flutter rhythm. Cardiologia Croatica. 2013;8.

231. Kuon E, Kreplin M, Weiss W, Dahm JB. The challenge presented by right atrial myxoma. Herz. 2004;29(7):702.

232. Kuralay E, Cingöz F, Günay C, Demirkiliç U, Tatar H. Huge right atrial myxoma causing fixed tricuspid stenosis with constitutional symptoms. Journal of Cardiac Surgery. 2003;18(6):550–3.

233. Kurnicka K, Domienik-Karłowicz J, Ciurzyński M, Biederman A, Pruszczyk P. Right atrial myxoma with pulmonary embolism. Kardiologia Polska (Polish Heart Journal). 2015;73(4):298–298.

234. Kuroda H, Nitta K, Ashida Y, Hara Y, Ishiguro S, Mori T. Right atrial myxoma originating from the tricuspid valve. J Thorac Cardiovasc Surg. 1995;109(6):1249–50.

235. Kuroda T, Yokoyama Y, Yuhara S, Okawa H, Hasegawa H, Yokote J, et al. Giant biatrial myxoma with two different gross findings. General Thoracic and Cardiovascular Surgery. 2018;66:358–60.

236. Kwon OY, Kim GJ, Jang WS, Lee YO, Cho JY, Lee JT. Fourth Recurrence of Cardiac Myxoma in a Patient with the Carney Complex. Korean J Thorac Cardiovasc Surg. 2016;49(2):119–21.

237. Kynta RL, Rawat S, Lyngdoh BS, Gunasekaran AK, Fanai V, Kapoor M, et al. Eustachian valve myxoma: a rare cause of Budd–Chiari syndrome. General Thoracic and Cardiovascular Surgery. 2021;69(8):1243–6.

238. Lamarche Y, Hébert Y, Leung TK, Perrault LP. Right atrial myxoma with vascular supply from right and left coronary vessels. European journal of cardio-thoracic surgery. 2007;32(3):532–532.

239. Layfield LJ, Dodd LG. Fine‐needle aspiration of a primary right atrial myxoma. Diagnostic cytopathology. 1996;14(2):162–4.

240. Lee WC, Chen HC, Chua S. Systemic embolism from bilateral atrial myxomas. Journal of Echocardiography. 2018;16(2):89–90.

241. Lempesis IG, Naxaki A, Koukoufiki E, Karagkouni I, Tzanatou A, Tourtidou C, et al. A right atrial myxoma presenting with misleading features of acalculous cholecystitis. Oxford Medical Case Reports. 2020;2020(2):omaa012.

242. Leonard S, Ryan J. A heavy heart; A massive right atrial myxoma causing fatigue and shortness of breath. Ir Med J. 2010;103(3):83–4.

243. Levin H, Cha SD, Sumathisena, Gonzalez-Lavin L, Gooch AS, Maranhao V. Detection of right atrial myxoma by coronary cinearteriography. Cathet Cardiovasc Diagn. 1986;12(6):414–6.

244. Lewitowicz P, Bernaczyk P, Horecka-Lewitowicz A, Leszczyńska U, Reszeć J, Hirnle T, et al. Ancient cardiac myxomas – another point of view in the light of tetraspanins. Polish Journal of Pathology. 2016;67(1):69–77.

245. Li H, Guo H, Xiong H, Xu J, Wang W, Hu S. Clinical features and surgical results of right atrial myxoma. Journal of Cardiac Surgery. 2015;31(1):15–7.

246. Liebler GA, Magovern GJ, Park SB, Cushing WJ, Begg FR, Joyner CR. Familial myxomas in four siblings. Journal of Thoracic and Cardiovascular Surgery. 1976;71(4):605–8.

247. Lin YH. Right atrial myxoma with pulmonary embolism. Cheng Ching Med J. 2007;3:7–11.

248. Liu D, Dong R. Clinical manifestation and surgical treatment analysis of five cases with biatrial myxoma. International Journal of Cardiology. 2016;228:309–12.

249. Liu Q, Zuo C, Lv T, Cui B. Pulmonary embolism caused by right atrial myxoma on FDG PET/CT. Clin Nucl Med. 2013;38(11):928–30.

250. Longatto FC, Santos T, Soares MJM, Negrisoli J, Leal T, Biselli B, et al. Upper vena cava syndrome secondary to giant atrial myxoma. Rev Assoc Med Bras (1992). 2018;64(12):1077–80.

251. Lopez N, Knight L, Slaton J. Right Atrial Myxoma: A Long Journey from Diagnosis to Surgery. 2020;

252. Lorentz MN, Vrandecic EC, Drumond LF, Soares RR. Right atrial myxoma associated with acute cor pulmonale. Case report. Rev Bras Anestesiol. 2008;58(1):69–72.

253. Low KB, Huang J, Lim CH. Clinics in diagnostic imaging (126). Right atrial myxoma. Singapore Med J. 2009;50(5):546–9; quiz 550.

254. Lyons HA, Kelly Jr JJ, Nusbaum N, Dennis C. Right atrial myxoma; a clinical study of a patient in whom diagnosis was made by angiocardiography during life; surgically removed. Am J Med. 1958;25(2):321–6.

255. Lyons SV, McCord J, Smith S. Asymptomatic giant right atrial myxoma: role of transesophageal echocardiography in management. Am Heart J. 1991;121(5):1555–8.

256. Ma G, Wang D, He Y, Zhang R, Zhou Y, Ying K. Pulmonary embolism as the initial manifestation of right atrial myxoma: A case report and review of the literature. Medicine (Baltimore). 2019;98(51):e18386–e18386.

257. Ma S, Xu Q, Shi R, Zhang X, Chen X. The omitted symptoms challenge the diagnosis of right atrial myxoma: a case report. BMC Cardiovasc Disord. 2020;20(1):149–149.

258. Ma X, Yan W, Guo X, Sun Z, Xie M. Right Coronary Artery-Right Atrial Fistula in Right Atrial Myxoma: A Rare Combination. Circ Cardiovasc Imaging. 2022;15(7):e014097–e014097.

259. Macoun SJ. Cardiac myxoma. Thorax. 1949;4(1):39–43.

260. Majumdar G, Agarwal S, Pande S, Tewari S. Right-atrial myxoma clinically mimicking recurrence of rheumatic valve disease long time after mitral valve repair. Indian Heart J. 2016;68 Suppl 2(Suppl 2):S135-s137.

261. Mallick SR, Das P, Shukla B, Kothari S, Devagourou V, Ray R. Right atrial myxoma with glandular differentiation: A rare entity in pediatric age group. Ann Pediatr Cardiol. 2010;3(2):159–62.

262. Manda GE, Mtekateka M, Kunkanga S, Kayange N. Unusual presentation of atrial Myxoma in a young Malawian male: Case report and review of literature. Malawi Med J. 2021;33(2):140–1.

263. Manduz S, Katrancioglu N, Karahan O, Yucel O, Yilmaz M. Diagnosis and follow up of patients with primary cardiac tumours: a single-centre experience of myxomas: cardiovascular topics. Cardiovascular Journal of Africa. 2011;22(6):310–2.

264. Marinakis S, Mircev D, Wauthy P. Cryoablation for a right atrial myxoma arising from the Koch’s triangle: a case report. Journal of Cardiothoracic Surgery. 2013;8:1–3.

265. Marks PH. Clinical manifestations of primary cardiac tumours. Cardiovascular Journal of South Africa. 1996;(SUPPL. 1):C12–6.

266. Marpole DG, Kloster FE, Bristow JD, Griswold HE. Atrial myxoma, a continuing diagnostic challenge. Am J Cardiol. 1969;23(4):597–602.

267. Martin CE, Hufnagel CA, de Leon Jr AC. Calcified atrial myxoma: diagnostic significance of the “systolic tumor sound” in a case presenting as tricuspid insufficiency. Am Heart J. 1969;78(2):245–50.

268. Martin LW, Wasserman AG, Goldstein H, Steinberg JS, Mills M, Katz RJ. Multiple Cardiac Myxomas with Multiple Recurrences: Unusual Presentation of a “Benign” Tumor. Annals of Thoracic Surgery. 1987;44(1):77–8.

269. Martinez MJ, Franco EH, Avalos LL, Perez AM. Multiple calcified right atrial myxomas associated with tricuspid insufficiency in a child. Cardiovascular Diseases. 1979;6(3):324–30.

270. Marvasti MA, Obeid AI, Potts JL, Parker FB. Approach in the management of atrial myxoma with long-term follow-up. Ann Thorac Surg. 1984;38(1):53–8.

271. Matsushita S, Kuramochi M, Kaneko J, Kuramoto K. Right atrial myxoma mimicking pericarditis. Phonocardiographic and hemodynamic consequences of intracardiac tumor movement. Jpn Circ J. 1968;32(9):1283–90.

272. McCoskey EH, Mehta JB, Krishnan K, Roy TM. Right atrial myxoma with extracardiac manifestations. Chest. 2000;118(2):547–9.

273. McGarry KM, Jugdutt BI, Rossall RE. The modern diagnosis of cardiac myxoma: role of two-dimensional echocardiography. Clin Cardiol. 1983;6(10):511–8.

274. Mehrotra D, Riordon J. Multiple intramural right atrial myxomas: Encountered incidentally during coronary artery bypass surgery. TEXAS HEART INSTITUTE JOURNAL. 2013;40(1):102–3.

275. Mendoza C, Bernstein E, Ferreira A. Multiple recurrences of nonfamilial cardiac myxomas: a report of two cases. Texas Heart Institute Journal. 2007;34(2):236.

276. Mendoza CE, Rosado MF, Bernal L. The role of interleukin-6 in cases of cardiac myxoma: clinical features, immunologic abnormalities, and a possible role in recurrence. Texas Heart Institute Journal. 2001;28(1):3.

277. Menoni K, Chitra S, Sulochana S, Muthusubramanian P, meenakshi D. A Case Series of Atrial Myxomas–A Tertiary Care Centre Experience. Journal of Pharmaceutical Research International. 2021;417–24.

278. Menti E, Gonzalez VL, Paula A, Osorio A, Cocco L. Right Atrial Myxoma: Rare Occurrence of an Uncommon Disease. Arq Bras Cardiol: Imagem Cardiovasc. 2016;29(2):63–6.

279. Merli VN, Dell’Oglio S, Grazioli V, Monterosso C, Vanini B, Gori M, et al. Surgical treatment for pulmonary embolization of a right atrial myxoma. The Annals of Thoracic Surgery. 2019;107(4):e245–6.

280. Meyers SN, Shapiro SE, Barresi V, DeBoer AA, Pavel DI, Gracey DR, et al. Right atrial myxoma with right to left shunting and mitral valve prolapse. Am J Med. 1977;62(2):308–14.

281. Mielczarek A, Szymczyk E, Religa G, Kaszczyński T, Lipiec P, Wierzbowska-Drabik K, et al. Right atrial myxoma as an atypical source of pulmonary embolism. Folia Cardiologica. 2022;17(2):131–3.

282. Milgalter E, Lotan H, Schuger L, Ben-Horin Y, Uretzky G, Appelbaum A, et al. Cardiac myxomas - Surgical experience with a multi-faceted tumor. THORACIC AND CARDIOVASCULAR SURGEON. 1987;35(1):115–8.

283. Miller DV, Tazelaar HD, Handy JR, Young DA, Hernandez JC. Thymoma arising within cardiac myxoma. Am J Surg Pathol. 2005;29(9):1208–13.

284. Miller GA, Paneth M, Gibson RV. Right atrial myxoma with right-to-left interatrial shunt and polycythaemia. Br Med J. 1968;3(5617):537–8.

285. Min SY, Lim YH, Lee HT, Shin J, Kim KS, Kim H. Biatrial myxoma and multiple organ infarctions combined with Leriche syndrome in a female patient. BMC Cardiovascular Disorders. 2014;14:1–4.

286. Minhas S. RIGHT ATRIAL MYXOMA COMPLICATED BY PULMONARY EMBOLISM AND ACUTE CONGESTIVE HEART FAILURE: RARE CASE REVIEW. Chest. 2020;158(4):A2136.

287. Mittle S, Makaryus AN, Boutis L, Hartman A, Rosman D, Kort S. Right-sided myxomas. Journal of the American Society of Echocardiography. 2005;18(6):695–695.

288. Miyauchi Y, Endo T, Kuroki S, Hayakawa H. Right atrial myxoma presenting with recurrent episodes of pulmonary embolism. Cardiology. 1992;81(2–3):178–81.

289. Modi K, Pullen J, Reddy P. Right atrial myxoma with atrial septal defect: A case report and review of literature. Internet Journal of Cardiology [Internet]. 2009;6(2). Available from: https://www.scopus.com/inward/record.uri?eid=2-s2.0-77953395409&partnerID=40&md5=9610f1401be29405a05e30f9dae322d2

290. Mohammad Karimi V, Anushiravani A, Dabbaghmanesh MH, Hosseinzadeh M, Rasekhi AR, Zamirian M. Myxoma immediately above the junction of the inferior vena cava and the right atrium: A rare cause of Budd-Chiari syndrome. Journal of Tehran University Heart Center. 2016;11(3):139–42.

291. Molnar A, Encică S, Săcui DM, Mureşan I, Trifan AC. A very rare association between giant right atrial myxoma and patent foramen ovale. Extracellular matrix and morphological aspects: a case report. Rom J Morphol Embryol. 2016;57(2):573–7.

292. Mori K, Oonaka M, Tanaka T, Takeda R. Prolapsing right atrial myxoma. Cardiology. 1979;64(1):58–63.

293. Moritz HA, Azad SS. Right atrial myxoma: case report and anaesthetic considerations. Can J Anaesth. 1989;36(2):212–4.

294. Moriuchi M, Saito S, Kamata T, Takaiwa Y, Tanigawa N, Honye J, et al. Contrast echocardiography in a patient with right atrial myxoma. International Journal of Angiology. 1998;7(2):115–7.

295. Morrissey JF, Campeti FL, Mahoney EB, Yu PN. RIGHT ATRIAL MYXOMA. REPORT OF TWO CASES AND REVIEW OF THE LITERATURE. Am Heart J. 1963;66:4–14.

296. Mudgalkar N, Reddy KR, Kumar KS, Swaroopa V. Management of Right atrial myxoma–Anesthesia perspective. 2018;

297. Munirathinam GK, Kumar B, Singh H. Right atrial myxoma with pulmonary artery hypertension: role of transesophageal echocardiography in detection of cause and perioperative management. Journal of Cardiothoracic and Vascular Anesthesia. 2018;32(2):801–6.

298. Murayama H, Tamaki S, Kato N, Yuji N, Yokote J, Mutsuga M, et al. Right atrial myxoma associated with atrial septal defect: a case report and review of the literature. Ann Thorac Cardiovasc Surg. 2001;7(3):166–9.

299. Muroff LR, Johnson PM. Right atrial myxoma presenting as nonresolving pulmonary emboli: case report. J Nucl Med. 1976;17(10):890–2.

300. Muthiah R. Right atrial Myxoma—A case report. Case Reports in Clinical Medicine. 2016;5(03):71.

301. Nakabayashi K, Murata S, Kato H, Oka T. The differentiation of giant right atrial myxoma from metastatic cancer with the use of multiple imaging modalities. Internal Medicine. 2016;55(8):925–8.

302. Nakamura K, Asai T, Murakami M, Saito Y, Yoshimoto A, Yamaguchi H. Giant right atrial myxoma associated with tricuspid regurgitation. Jpn J Thorac Cardiovasc Surg. 2006;54(8):332–4.

303. Nakayama M, Matsumura K, Abe I, Kaku R, Kobayashi K, Fujishima M, et al. Invasive Development of Right Atrial Myxoma— A Case Report. Angiology. 1993;44(9):739–44.

304. Naqshband MS, Abid AR, Akhtar RP, Ayub M, Khan JS. Biatrial myxoma and atrial septal defect. J Coll Physicians Surg Pak. 2010;20:202–4.

305. Nardi C, De Carlo M, Milano A, Bortolotti U. The wrecking ball effect of a right atrial myxoma. European journal of cardio-thoracic surgery. 2000;17(3):338–338.

306. Naser N, Hadziomerovic N, Bahram D, Kacila M, Pandur S. Giant Right Atrial Myxoma with Symptoms of Right Heart Failure. Med Arch. 2021;75(1):66–8.

307. Nasser WK, Davis RH, Dillon JC, Tavel ME, Helmen CH, Feigenbaum H, et al. Atrial myxoma. II. Phonocardiographic, echocardiographic, hemodynamic, and angiographic features in nine cases. Am Heart J. 1972;83(6):810–23.

308. Natarajan P, Vijayanagar RR, Eckstein PF, Bognolo DA, Toole JC. Right atrial myxoma with atrial septal defect: a case report and review of the literature. Cathet Cardiovasc Diagn. 1982;8(3):267–72.

309. Nath D, Arava S, Ray R, Bhoje AK, Saxena R, Chaudhary SK. Familial biatrial cardiac myxoma with glandular elements: A rare entity with review of literature. Indian Journal of Pathology and Microbiology. 2017;60(4):568.

310. Natraj Setty NH, Yeriswamy M, Jadav S, Kharge J, Raghu T, Patil R, et al. Cricket ball in the right heart: a case report of right atrial myxoma. Annals of Medicine and Surgery. 2019;48:39–42.

311. Nawaz H, Changal K, Hariri I, Idris O, Bhatti K. Asymptomatic spontaneous complete dislodgement of a right atrial myxoma. British Journal of Hospital Medicine. 2019;80(5):ii–ii.

312. Nazzi M, Belhachmi H, Badidi M, Zbir E, Mohty D. A voluminous right atrial myxoma revealed by right side heart failure. Echocardiography. 2013;30(3):E89–91.

313. Niarchos C, Frangides C, Kouni SN, Kounis NG. Ascites and other extracardiac manifestations associated with right atrial myxoma: a case report. Angiology. 2005;56(3):357–60.

314. Nikyar AR. Right atrial myxoma. Medical Journal of the Islamic Republic of Iran. 1989;3(1–2):87–9.

315. Nili M, Arditi S, Halevi A, Levy MJ. Atrial tumors--surgical experience of 9 cases. Scand J Thorac Cardiovasc Surg. 1983;17(2):93–100.

316. Nina VJ, Silva NA, Gaspar SF, Rapôso TL, Ferreira EC, Nina RV, et al. Atypical size and location of a right atrial myxoma: a case report. J Med Case Rep. 2012;6:26–26.

317. Nivargi V, Durairaj M, Makhale C. Right Atrial Myxoma. J Assoc Physicians India. 2017;65(5):87–87.

318. Nolan J, Carder PJ, Bloomfield P. Atrial myxoma: tumour or trauma? Br Heart J. 1992;67(5):406–8.

319. Northcote RJ, Ballantyne D, Sethia B. Delay in diagnosis of right atrial myxoma. Clinical Cardiology. 1985;8(2):107–10.

320. Numaguchi R, Hashimoto M, Koshima R, Mitsube K. Surgical treatment of a giant right atrial myxoma. Surgical Case Reports. 2020;6:1–3.

321. Obagi A, Desai D, Mazahir U, Johnson D, Berger L. Large right atrial myxoma presenting as bilateral pulmonary embolism. Cureus. 2021;13(6).

322. Ohshima H, Kawashima E, Ogawa Y, Tobise K, Onodera S. Demonstration of the inner structure of a right atrial myxoma by transoesophageal echocardiography. Eur Heart J. 1993;14(1):132–4.

323. Ojji DB, Ajiduku SS, Omonua OO, Abdulkareem LL, Parsonage W. A probable right atrial myxoma prolapsing through the tricuspid valve into the right ventricle: a case report. Cases J. 2008;1(1):386–386.

324. Oldershaw PJ, Sutton StJ M, Gibson RV. Long asymptomatic period of atrial myxomas. Thorax. 1980;35(1):70–1.

325. Omar H. The value of coronary angiography in the work-up of atrial myxomas. Herz. 2013;40(3):442.

326. Onan B, Bayramoğlu Z, Onan IS, Akpınar B. Right atrial myxoma associated with portal and splenic vein thrombosis in a patient with Budd-Chiari syndrome. Turk Kardiyol Dern Ars. 2011;39(4):320–4.

327. Oommen MJ, Luqman N, Kafeel G, Chin Kah T, Chong CF. Right atrial myxoma: An unusual cause of clinical right heart failure. Brunei International Medical Journal. 2012;8(1):52–5.

328. Oshiumi M, Hashimoto K, Sasaki T, Takakura H, Hachiya T, Onoguchi K. Right atrial myxoma complicated with pulmonary embolism. Jpn J Thorac Cardiovasc Surg. 2001;49(7):449–52.

329. Oumar K Coulibaly Bréhima, Sangaré Ibrahima, Doumbia Coumba, Thiam SS Camara Youssouf, Coulibaly Souleymane, Diallo Souleymane, Touré, Mamadou MI Diall Io Bella, Diarra Mamadou Bocary. Right Atrial Myxoma Versus Highblood Pressure And Coronary Artery Disease In The Development Of Chest Pain And Dyspnoea. International Journal Of Medical Science And Clinical Inventions. 2016;

330. Ozer N, Deveci OS, Okutucu S, Demircin M. Asymptomatic right atrial myxoma originating from the inferior vena cava and right atrium junction in a patient with breast ductal adenocarcinoma. Turk Kardiyol Dern Ars. 2009;37(7):479–82.

331. Pandey AC, Carey JJ, Thompson JL. Right atrial myxoma presenting as a pulmonary embolism in a 32-year-old female. JRSM Cardiovascular Disease. 2019;8:2048004018817606.

332. Papadopoulos K, Alexiou C, Ozden Tok O, Vannan MA. Intraoperative embolism of a right atrial myxoma: a case report. Eur Heart J Case Rep. 2020;4(6):1–4.

333. Paquet E. The diagnostic approach to auricular myxomas. Canadian Medical Association Journal. 1956;74(2):121.

334. Park JM, Garcia RR, Patrick JK, Waagner D, Anuras S. Right atrial myxoma with a nonembolic intestinal manifestation. Pediatr Cardiol. 1990;11(3):164–6.

335. Parsons AM, Detterbeck FC. Multifocal right atrial myxoma and pulmonary embolism. The Annals of thoracic surgery. 2003;75(4):1323–4.

336. PASAOGLU I, DEMIRCIN M, ÖZKUTLU S, BOZER AY. Right atrial myxoma in an infant. Japanese heart journal. 1990;32(2):263–6.

337. Patrianakos AP, Parthenakis FI, Nyktari E, Kochiadakis GE, Koutsopoulos AV, Vardas PE. Right atrial myxoma: echocardiographic appearance. Eur J Echocardiogr. 2008;9(3):422–3.

338. Pehlivan S, Akçan R, Yıldırım MŞ, Gökmen A, Yöndem M. Atypical location of extracardiac myxoma: A case report. European Journal of General Medicine. 2017;14(3):76–8.

339. Peregud-Pogorzelska M, Lewandowski M, Trzcińska-Butkiewicz B, Brzosko M. Right atrial myxoma: a potential accelerator of pulmonary hypertension in the course of systemic sclerosis. The role of interleukin-6. Advances in Dermatology and Allergology/Postępy Dermatologii i Alergologii. 2019;36(3):354–7.

340. Pernod J, Piwnica A, Duret JC. Right atrial myxoma: an echocardiographic study. Br Heart J. 1978;40(2):201–3.

341. Pillai JB, Feindel CM, Butany J, Murphy P. A diagnostic challenge - An unusual right atrial mass, 12 years following atrial septal defect surgery. Interactive Cardiovascular and Thoracic Surgery. 2005;4(4):285–6.

342. Pliakos C, Alexiadou E, Metallidis S, Papavramidis TS, Kapoulas S, Sapalidis K, et al. Right atrium myxoma coexisting with antiphospholipid syndrome: a case report. Cardiovascular Ultrasound. 2009;7(1):1–4.

343. Pores IH, Abel RM, Gray L, Jacobs GP. Giant right atrial myxoma with rheumatic mitral valve disease. Angiology. 1984;35(5):313–9.

344. Powers JC, Falkoff M, Heinle RA, Nanda NC, Ong LS, Weiner RS, et al. Familial cardiac myxoma: emphasis on unusual clinical manifestations. J Thorac Cardiovasc Surg. 1979;77(5):782–8.

345. Pussadhamma B, Wongbuddha C. An extensively calcified right atrial myxoma. The Annals of Thoracic Surgery. 2015;100(2):731.

346. Puvaneswary M, Thomson D. Magnetic resonance imaging features of an infected right atrial myxoma. Australas Radiol. 2001;45(4):501–3.

347. Quashem M, ROYb HR, Kibria MG, ISLAM AM, SHAFI A, MAJUMDERe N, et al. Large Right Atrial Myxoma-An Uncommon Cardiac Tumor Needs Urgent Surgery. Journal of Bangladesh College of Physicians and Surgeons. 2009;27(1):52.

348. Rafiq I, Parthasarthy H, Clark CG. Atrial myxoma’s and coronary angiography. Catheterization and Cardiovascular Interventions. 2010;76(1):156–7.

349. Ragland MM, Tak T. Detection of atypical right atrial myxoma by echocardiography. Cardiol Rev. 2006;14(2):99–100.

350. Rajani M, Mukhopadhyay S, Dogra B, Shrivastava S, Bhatia ML, Venugopal P, et al. Primary Cardiac Mass Lesions (A Study of 24 Cases). Australasian Radiology. 1988;32(1):84–91.

351. Rajani R, Sarangmath N, Mishra B. Massive right atrial myxoma. International journal of cardiology. 2008;128(1):121–2.

352. Ramsdale DR, Green GJ, Charles RG. Abnormal diastolic movement of the interventricular septum caused by a prolapsing right atrial myxoma. Br Heart J. 1986;56(6):569–71.

353. Ran H, Chen G, Hu J, He Y, Liu J, Li F, et al. Case report: biatrial myxoma with pulmonary embolism and cerebral embolism: clinical experience and literature review. Frontiers in Cardiovascular Medicine. 2022;9:812765.

354. Randriamanga RL, Rakotomijoro E, Rakotonirina MBA. Repeated discomfort leading to a diagnosis of a voluminous right atrial myxoma in an adolescent patient: a case report. Pan African Medical Journal. 2022;41(1).

355. Rao VR, Jagannath K, Sunil PK, Madhusudana N. A rare disappearing right atrial mass. Interactive cardiovascular and thoracic surgery. 2012;15(2):290–1.

356. Rathor DKS, Jhajhria NS, Gupta V. Atypical clinical presentation of right atrial myxoma: a case report. International Surgery Journal. 2017;4(4):1444–6.

357. Reber D, Birnbaum DE. Recurrent cardiac myxoma: why it occurs. A case report with literature review. J Cardiovasc Surg (Torino). 2001;42(3):345–8.

358. Rey M, Tuñon J, Compres H, Rabago R, Fraile J, Rabago P. Prolapsing right atrial myxoma evaluated by transesophageal echocardiography. Am Heart J. 1991;122(3 Pt 1):875–7.

359. Reynen K, Köckeritz U. Right atrial myxoma. Clinical research in cardiology. 2006;95(1):3.

360. Riad M, Thangathurai D, Farhoomand L. Infected atrial myxoma presenting with septic shock. ANESTHESIA AND ANALGESIA-CLEVELAND-. 2005;98(4; SUPP):SCA 128-SCA 128.

361. Richkind KE, Wason D, Vidaillet HJ. Cardiac myxoma characterized by clonal telomeric association. Genes, Chromosomes and Cancer. 1994;9(1):68–71.

362. Ridge CA, Killeen RP, Sheehan KM, Ryan R, Mulligan N, Luke D, et al. Giant right atrial myxoma: characterization with cardiac magnetic resonance imaging. Clinical imaging. 2010;34(3):231–3.

363. Roberts DH, Ramsdale DR. First degree atrioventricular block associated with right atrial myxoma. Postgrad Med J. 1990;66(772):140–1.

364. Roberts N. Mimics of bacterial endocarditis. The American Journal of Cardiology. 1970;26(5):528–31.

365. Roguin N, Amikam S, Riss E. Prolapsing right atrial myxoma: clinical and heamodynamic considerations. Br Heart J. 1977;39(5):577–80.

366. Rokadia HK, Heresi GA, Tan CD, Raymond DP, Budd GT, Farver C. A 33-year-old man with multiple bilateral pulmonary pseudoaneurysms. Chest. 2015;148(4):e112–7.

367. Romanović R, Ratković N, Davičević Ž, Ilić R. Massive right atrial myxoma with dyspnea at rest in an elderly patient: A case report. Vojnosanitetski pregled. 2015;72(3).

368. Romeo R, Maugeri C, Ragusa A, Romeo A, Maugeri D, Sorace R. Massive right atrial myxoma: An unusual presentation in an elderly patient. Archives of Gerontology and Geriatrics. 2012;55(1):143–4.

369. Roudaut R, Pouget B, Videau P, Clementy J, Choussat A, Baudet E, et al. Right atrial myxoma in an asymptomatic child. Echocardiographic diagnosis. European Heart Journal. 1980;1(6):453–9.

370. Saadeh AM, Hijazi EM, Saadeh NA. Right atrioventricular myxoma presenting with recurrent syncopal attacks. The American Journal of Case Reports. 2021;22:e927874-1.

371. Sabageh D, Odujoko OO, Komolafe AO. Right atrial myxoma as a possible cause of hemorrhagic stroke and sudden death. Nigerian Medical Journal: Journal of the Nigeria Medical Association. 2012;53(2):102.

372. Sabzi F, Nasiri B. Myxoma of the superior vena cava origin presented as a right atrial mass. The Journal of Tehran University Heart Center. 2012;8(4):202.

373. Saha D, Sinha L, Samal S, Sharma P, Naqvi SEH, Geelani MA. An unusual presentation of right atrial myxoma: A case report. Oman Medical Journal. 2021;38(1):e466.

374. Sahin V, Uyar IS, Evrengul H, Erturk A, Ates M. Massive right atrial myxoma causing right ventricular inflow obstruction: an unusual presentation of myxoma. Journal of Cardiac Surgery. 2010;25(3):293–5.

375. Sahitya V, Anandam G, Shastry S. Right atrial myxoma in a 65-year-old female: A rare presentation. Medical Journal of Dr DY Patil Vidyapeeth. 2018;551–3.

376. Saitoh H, Kubota H, Takeshita M, Mizuno A, Suzuki M. Right atrial myxoma with right to left shunt and coronary artery disease. Jpn Circ J. 1994;58(1):76–9.

377. Sajja LR, Mannam GC, Krishnam Raju P, Sriramulu S, Rajasekhar A. Right atrial myxoma arising from crista terminalis in septuagenarian. Asian Cardiovascular and Thoracic Annals. 2001;9(4):322–4.

378. Salehi R, Pourafkari L, Shokouhi B, Nader ND. Case images: Right atrial myxoma causing distortion of interventricular septum in diastole. Turk Kardiyol Dern Ars. 2015;43(5):494–494.

379. Salehi R, Chenaghlou M, Mohamadi A, Javanshir E. Cardiac Myxoma Arising From the Superior Vena Cava: A Case Report. Iranian Heart Journal. 2022;23(3):131–4.

380. Šaler F, Bodrožić-Džakić Poljak T, Blažeković R, Ćatić J, Manola Š. Asymptomatic patient with giant right atrial myxoma. Cardiologia Croatica. 2022;17(9–10):296–296.

381. Sánchez-Sotelo VM, Velázquez-Sotelo CE, Vega-Hernández R, Mejía-Bañuelos RM. Right atrial heart myxoma, two different presentations of the same entity and histopathological findings. Cardiovascular and metabolic science. 2022;33(3):106–12.

382. Sangodkar S, Schevchuck A. RIGHT ATRIAL MYXOMA AS A CAUSE OF STROKE. Journal of the American College of Cardiology. 2016;67(13S):1207–1207.

383. Sanjeev OP, Nath SS, Malviya D, Rajput SS. Right atrial myxoma: Unusual location; uncommon association. Annals of cardiac anaesthesia. 2018;21(4):437.

384. Sannerstedt R, Varnauskas E, Paulin S, Linder E, Ljunggren H, Werko L. Right atrial myxoma. Report of a case and review of the literature. Am Heart J. 1962;64:243–54.

385. Sansone F, Ceresa F, Patanè F. Two cases of right atrial myxoma in redo patients. A mere coincidence? G Chir. 2013;34(1–2):11–3.

386. Santos-Ortega A, Sambola A, Martí G, Barrabés JA, Rodríguez R, García-Dorado D. Giant Right Atrial Myxoma and Refractory Hypoxia: An Unexpected Combination. Revista Espanola de Cardiologia (English ed). 2016;69(12):1219–20.

387. Sanyal SK, de Leuchtenberg N, Rojas RH, Stansel H, Browne MJ. Right atrial myxoma in infancy and childhood. The American Journal of Cardiology. 1967;20(2):263–9.

388. Şaşkın H, Düzyol Ç, Özcan KS, Aksoy R. Right atrial myxoma mimicking tricuspid stenosis. BMJ Case Rep. 2015;2015.

389. Sato H, Tanaka T, Kasai K, Kita T, Tanaka N. Sudden death due to acute pulmonary embolism from asymptomatic right atrial myxoma. Journal of Forensic and Legal Medicine. 2008;15(7):454–6.

390. Sato I, Kanda H, Kunisawa T. Utility of x-plane TEE imaging in giant right atrial myxoma. Journal of Anesthesia. 2016;30:363–363.

391. Sato T, Watanabe H, Okawa M, Iino T, Iino K, Ishibashi K, et al. Right atrial giant myxoma occupying the right ventricular cavity. The Annals of thoracic surgery. 2012;94(2):643–6.

392. Schmid C, Paterakis S, Likungu JA, Kirchhoff PG. Cardiac myxoma. Clinical and pathologic features in 15 cases. Klin Wochenschr. 1988;66(16):713–7.

393. Schwab J, Haack G, Bär I, Nagel E, Zahn R. Giant right atrial myxoma verified by cardiovascular magnetic resonance. Herz. 2007;32(5):430–1.

394. Scohy TV, Lecomte PV, McGhie J, Meijer R, Gommers D, Hofland J, et al. Intraoperative real time three-dimensional transesophageal echocardiographic evaluation of right atrial tumor. Echocardiography. 2008;25(6):646–9.

395. Seifert P, Chomka EV, Stagl R, Swarner D, Brundage BH, Levitsky S. Application of the cine computed tomographic scan for precise localization of the origin of an atrial myxoma: Surgical implications. The Annals of thoracic surgery. 1986;42(4):469–70.

396. Sellke FW, Lemmer Jr JH, Vandenberg BF, Ehrenhaft JL. Surgical treatment of cardiac myxomas: Longterm results. The Annals of Thoracic Surgery. 1990;50(4):557–61.

397. Selvaganesan S, Khidr SS, Biederman RWW. Calcified mass in the right atrium extending into the inferior vena cava with pulmonary artery embolization. Typical or atypical myxoma? Echocardiography. 2020;37(7):1130–3.

398. Selvaraj A, Kumar R, Ravikumar E. Surgical management of right atrial myxomas. A 15 year experience with review of the literature. J Cardiovasc Surg (Torino). 1999;40(1):101–5.

399. Shaikh AH, Khan G, Hanif B, Malik F, Bashir A. Biatrial myxoma. J Coll Physicians Surg Pak. 2008;18:639–40.

400. shamim Rahman MS, Michael H. A rare presentation of chest pain and syncope: massive right atrial myxoma. Postgrad Med J. 2012;88(1045):671–2.

401. Sharifkazemi M, Rezaian G, Abtahi F. Right Atrial Myxoma and Chronic Transudative Ascites: A Rare and Challenging Clinical Presentation. CASE (Phila). 2018;2(4):120–2.

402. Sharma D, Dorgan E, Douglas H, Trouton T, McMullan R, Parissis H. Aspergillus infection in pulmonary cavitating lesions with right atrial myxoma. Asian Cardiovasc Thorac Ann. 2014;22(9):1090–2.

403. Sheriff EA, Vaidyanathan S, Chandran DK, Chidambaram K, Kalidas L, Sethuratnam R. Atrial myxoma invading right ventricular cavity and pulmonary artery. Int J Res Med Sci. 2017;5:3723–5.

404. Shetty Roy AN, Radin M, Sarabi D, Shaoulian E. Familial recurrent atrial myxoma: Carney’s complex. Clin Cardiol. 2011;34(2):83–6.

405. Shevchenko Y, Borshchev G. Off-pump Surgical Removal of Right Atrial Myxoma Without Hypothermia. 2020;

406. Shimizu M, Okuri H, Yokoyama K, Kawada H, Takizawa T, Kikawada R. A case of right atrial myxoma--effect of large myxoma in the right atrium studied by M-mode and Doppler echocardiography. Jpn Circ J. 1995;59(8):579–86.

407. Sievers J, Areskog NH, Stenport G. Right atrial myxoma with severe cyanosis. Cardiology. 1970;55(1):55–62.

408. Siltanen P, Tuuteri L, Norio R, Tala P, Ahrenberg P, Halonen PI. Atrial myxoma in a family. Am J Cardiol. 1976;38(2):252–6.

409. Silva D, Trautmann N, Rieß FC. Large Right Atrial Myxoma after Transcatheter Ablation. The Thoracic and Cardiovascular Surgeon. 2019;67(S 01):DGTHG-KV32.

410. Singh B, Gupta RK, Tandon R, Soni A, Kaur H, Mohan B, et al. Prolapsing giant right atrial myxoma in a young male presenting as pulmonary embolism. Echocardiography. 2022;39(4):645–6.

411. Singh S, Tripathy MP, Mohanty BB, Biswas S. Sporadic multicentric right atrial and right ventricular myxoma presenting as acute pulmonary thromboembolism. Heart Views: The Official Journal of the Gulf Heart Association. 2016;17(1):19.

412. Singhal SK, Aiyer P, Grover V, Gupta VK. Atypical size and location of a right atrial myxoma: a case report. International Surgery Journal. 2017;4(6):2073–6.

413. Sinha VK, Nair M. Giant right atrial myxoma—a rare case report and review of literature. INDIAN JOURNAL OF THORACIC AND CARDIOVASCULAR SURGERY. 2017;33(4):325–7.

414. Siraj N, Siraj M, Khanum S. Coronary artery disease and right atrial myxoma: A case report. Bangladesh J Radiol Imaging. 2008;16:34–5.

415. Skamrov A, Nechaenko M, Goryunova L, Feoktistova E, Khaspekov G, Kovalevsky D, et al. Gene expression analysis to identify mRNA markers of cardiac myxoma. Journal of molecular and cellular cardiology. 2004;37(3):717–33.

416. Smith ST, Hautamaki K, Lewis Jr JW, Serwin J, Alam M. Transthoracic and transesophageal echocardiography in the diagnosis and surgical management of right atrial myxoma. Chest. 1991;100(2):575–6.

417. Sommariva L, Auricchio A, Polisca P, Penta de Peppo A, Chiariello L. Right atrial myxoma with atypical features of syndrome myxoma. Am Heart J. 1993;126(1):256–8.

418. Srivastava S, Kisku N, Minhas H, Batra V, Raja N. Right Atrial Myxoma With Blood Supply From Left Circumflex Artery− A Rare Variant. MAMC Journal of Medical Sciences. 2017;3(1):31–3.

419. Stern MJ, Cohen MV, Fish B, Rosenthal R. Clinical presentation and non-invasive diagnosis of right heart masses. Br Heart J. 1981;46(5):552–8.

420. Storch HD. Right atrial myxomas. Chest. 1991;99(1):264–264.

421. Straw S, Sengupta A, Gantenby VK, Schlosshan D, Ferrara A, Witte KK. An enormous right atrial myxoma highlights the role of echocardiography in heart failure pathways: a case report. European Heart Journal-Case Reports. 2022;6(2):ytac042.

422. Strecker T, Agaimy A, Zelzer P, Weyand M, Wachter DL. Incidental finding of a giant asymptomatic right atrial tumor. International Journal of Clinical and Experimental Pathology. 2014;7(7):4528.

423. Subban V, Lakshmanan A, Sethurathinam R, Ajit MS. Right atrial myxoma--an unusual cause of pulmonary embolism. J Card Surg. 2012;27(5):604–604.

424. Sugeng L, Sahoo S, Lang RM. Atypical cardiac myxomas. Echocardiography. 2004;21(1):43–7.

425. Sultan FA, Hussain B, Rasheed F. Right atrial myxoma in a patient with Budd‑Chiari syndrome (RCD code: VI‑1A.1). Journal of Rare Cardiovascular Diseases. 2018;3(6):218–20.

426. Surabhi SK, Fasseas P, VanDecker WA, Hanau CA, Wolf NM. Right atrial myxoma in a patient presenting with syncope. Texas Heart Institute Journal. 2001;28(3):228.

427. Suzuki I, Koide S, Odagiri S, Shohtsu A. Right atrial myxoma developing 4 years following patch closure of an atrial septal defect: report of a case. Surg Today. 1994;24(2):176–8.

428. Suzuki JI, Takayama K, Mitsui F, Kono T, Yazaki Y, Takei M, et al. In situ interleukin-6 transcription in embryonic nonmuscle myosin heavy chain expressing immature mesenchyme cells of cardiac myxoma. CARDIOVASCULAR PATHOLOGY. 2000;9(1):33–7.

429. Symbas PN, Hatcher Jr CR, Gravanis MB. Myxoma of the heart: clinical and experimental observations. Ann Surg. 1976;183(5):470–5.

430. Taber RE, Lam CR. Diagnosis and surgical treatment of intracardiac myxoma and rhabdomyoma. The Journal of Thoracic and Cardiovascular Surgery. 1960;40(3):337–54.

431. Tagawa T, Okuda M, Sakuraba S. Anesthetic management of a patient with giant right atrial myxoma. Journal of Cardiothoracic and Vascular Anesthesia. 2010;24(3):532–3.

432. Talley RC, Baldwin BJ, Symbas PN, Nutter DO. Right atrial myxoma. Unusual presentation with cyanosis and clubbing. Am J Med. 1970;48(2):256–60.

433. Tang Y, Li J, Zhao F, Chen T. Total thoracoscopic surgery for biatrial cardiac myxoma: a case report. Annals of Translational Medicine. 2020;8(23).

434. Tataroǧlu C, Kirdar S, Döǧer F, Çetín N, Ceyhan C, Boǧa M. Glandular cardiac myxoma with associated human papilloma virus infection: Case report. Turkiye Klinikleri Cardiovascular Sciences. 2013;25(2):99–104.

435. Tatebe S, Ohzeki H, Miyamura H, Hayashi J, Hiratsuka M, Sunami E, et al. Carney’s complex in association with right atrial myxoma. Ann Thorac Surg. 1994;58(2):561–2.

436. Tok M, Oc M, Ucar H, Dogan O, Ozyuksel A, Kaya B, et al. Giant right atrial myxoma mimicking hepatic cirrhosis: a case report. In 2007. p. E107.

437. Tondelli M, Mandrioli J, Ficarra G, Pentore R, Girolami F, Ghidoni I, et al. Teaching NeuroImage: When right atrial myxoma meets patent foramen ovale: a case of paradoxical brain embolism. Neurology. 2008;70(1):e1-2.

438. Turlapati RV, Jacobs LE, Kotler MN. Right atrial myxoma causing total destruction of the tricuspid valve leaflets. Am Heart J. 1990;120(5):1227–31.

439. Tyson N, Efthymiou C. Unilateral finger clubbing as an unusual manifestation of right atrial myxoma. European Journal of Cardio-Thoracic Surgery. 2020;58(3):658–658.

440. Umana E, Alpert MA, Massey CV, Tucker JA, Damrich ME. Biatrial myxoma resembling an interatrial clot in transit on echocardiogram. Southern medical journal. 1999;92(10):1019–22.

441. Umeda Y, Matsuno Y, Imaizumi M, Mori Y, Iwata H, Takiya H. Right atrial myxoma with tumor vascularity originated from the left and right coronary arteries. International Journal of Cardiology. 2007;131(3):e137–9.

442. Umezawa S, Obayashi T, Chun YH, Kataoka N, Kanayama M, Takamoto T, et al. A large myxoma of the right atrium demonstrated by thallium-201. Ann Nucl Med. 1989;3(2):95–8.

443. Ungerman E, Haft W. Giant Right Atrial Myxoma: The Importance of Transesophageal Echocardiography during Diagnosis, Evaluation, and Resection. Journal of Perioperative Echocardiography. 2016;4(2):74–8.

444. Usman M, Umar H, Oboirien I, Isezuo S. A probable case of right atrial myxoma presenting with features of restrictive cardiomyopathy. 2019;

445. Vargas G, Fuentes Mendoza J, Ruiz Esparza M. P234 Giant right atrial myxoma presenting as right heart failure. European Heart Journal-Cardiovascular Imaging. 2020;21(Supplement_1):jez319. 099.

446. Vassiliadis N, Vassiliadis K, Karkavelas G. 1. Sudden Death Due to Cardiac Myxoma. Medicine, Science and the Law. 1997;37(1):76–8.

447. Vega J, Gabrielli L, Córdova Alvestegui SE, Mc-Nab Martin PA, Saavedra Madariaga RA, Piñeiro M, et al. “Gigantic” biatrial myxoma with right heart functional impairment. 2018;

448. Velvet AJ, Parekh V, Khan W, Ahmed I. A case report of a large right atrial myxoma: the role of virtual consultations and imaging during the COVID-19 pandemic. Oxf Med Case Reports. 2022;2022(6):omac059–omac059.

449. Venkatesan K, Swaminathan N. A Case of Silent Right Atrial Myxoma. University Journal of Medicine and Medical Specialities. 2021;7(2).

450. Vilacosta I, Zamorano J, Ramos JM, RomÁN JAS, Camino A, Roca V, et al. Infected Myxomas:Report of a Case and Review of the Literature. Echocardiography. 1994;11(1):29–33.

451. VL W. Unusual aspects of intracavitary tumors of the heart. Dis Chest. 1965;47:669–71.

452. Vohra H, Phillips N, Nel L, Diprose P, Ohri S. Unidentified retained inferior vena cava myxoma detected by intra-operative trans-oesophageal echocardiography. The Thoracic and Cardiovascular Surgeon. 2010;58(04):248–50.

453. Walker A, Wilkinson J, Goiti J. Acute bicaval obstruction as a result of intracapsular haemorrhage in a right atrial myxoma: report of a case. European journal of cardio-thoracic surgery. 1997;11(4):779–81.

454. Wang LW, Granger EK. Obstructive right atrial myxoma in association with Carney complex. Heart, Lung and Circulation. 2013;22(6):450–1.

455. Waxler EB, Kawai N, Kasparian H. Right atrial myxoma: echocardiographic, phonocardiographic, and hemodynamic signs. Am Heart J. 1972;83(2):251–7.

456. Weiss SL, Russell HM, Lay A, Backer CL. Calcified right atrial myxoma in an adolescent. World Journal for Pediatric and Congenital Heart Surgery. 2011;2(3):523–5.

457. Wiedermann C, Reinisch N, Fischer‐Colbrie R, Vollmar A, Herold M, Knapp E. Proinflammatory cytokines in cardiac myxomas. Journal of internal medicine. 1992;232(3):263–5.

458. Willey RF, Matthews MB, Walbaum PR. An unusual case of large right atrial myxoma. Br Heart J. 1980;44(1):108–10.

459. Wilsher ML, Roche AH, Neutze JM, Synek BJ, Holdaway IM, Nicholson GI. A familial syndrome of cardiac myxomas, myxoid neurofibromata, cutaneous pigmented lesions, and endocrine abnormalities. Aust N Z J Med. 1986;16(3):393–6.

460. Xiao Z hua, Hu J, Zhu D, Shi Y kang, Zhang E yong. Tricuspid valve obstruction and right heart failure due to a giant right atrial myxoma arising from the superior vena cava. Journal of cardiothoracic surgery. 2013;8(1):1–4.

461. Xie X, Bai J. Right atrial myxoma induced right ventricular inflow obstruction. J Card Surg. 2018;33(3):137–8.

462. Xie X, Bai J, Li X. Right atrial myxoma. J Card Surg. 2018;33(7):388–90.

463. Xiong Q, Liao B, Yu F, Li X. Cardiac myxoma in the right atrium. J Card Surg. 2019;34(10):1086–7.

464. Yamamoto T, Yamashita K, Hagiwara H. Infected atrial myxoma presenting with purpura. CMAJ. 2021;193(10):E340–E340.

465. Yanagawa Y, Shimazaki H, Shima K, Isoda S, Maehara T. Atrial Myxoma Occurring 15 Years After Subtotal Resection of Cerebellar Hemangioblastoma—Case Report—. Neurologia medico-chirurgica. 2007;48(1):37–9.

466. Yanardag H, Tetikkurt C, Yanardag E, Bilir M, Keleş İ. A Case of Right Atrial Myxoma Presenting as Bilateral Pneumonic Infiltration. American Journal of Cardiology. 2018;121(8):e134.

467. Yang T, Tsai J, Chang C, Kuo J, Hung C. Giant right atrial myxoma with pulmonary trunk dislodgement causing intermittent tricuspid obliteration and clinical manifestations of right heart failure. Echocardiography. 2011;28(9):E183–6.

468. Yavari A, El-Mahy H, McWilliams ET. Right atrial mass in the context of recurrent non-Hodgkin’s lymphoma: atrial myxoma presenting with atrial flutter. BMJ Case Rep. 2009;2009.

469. Yazici M, Ozhan H, Tetik O, Kinay O, Ergene O. Isolated large right atrial myxoma manifested by syncope. Texas Heart Institute Journal. 2004;31(3):324.

470. Yen RS, Allen B, Ott R, Brodsky M. The syndrome of right atrial myxoma, spotty skin pigmentation, and acromegaly. Am Heart J. 1992;123(1):243–4.

471. Yeshvanth SK, Permi HS, Laxminarayana KP, Shrinivas T, Shetty JK. Two cases of eosinophilic variant chromophobe renal cell carcinoma, with a rare association of right atrial myxoma in one of them. J Lab Physicians. 2011;3(2):116–8.

472. Yilmaz M, Gurbuz O, Cengiz M. Multicentric cardiac myxoma treated with extended surgery. Journal of international medical research. 2006;34(3):331–4.

473. Yin L, Yu S si, Wu H long, Ren H bo, Gong L geng. An atypical right atrial myxoma with spontaneous rupture. International Heart Journal. 2016;57(2):262–4.

474. Younes M, Al-Dairy A, Albadr A. Right atrial myxoma detected in a child two months after open-heart surgery. Asian Cardiovascular and Thoracic Annals. 2019;27(7):584–6.

475. Yuan S, Shinfeld A, Raanani E. Tricuspid valve myxoma: a case report and a collective review of the literature. Journal of Cardiac Surgery. 2009;24(1):69–72.

476. Yuce M, Dagdelen S, Ergelen M, Eren N, Caglar N. A huge obstructive myxoma located in the right heart without causing any symptom. International journal of cardiology. 2007;114(3):405–6.

477. Yuste P, Asin E, Cerdan FJ, de la Fuente A. Echocardiogram in right atrial myxoma. Chest. 1976;69(1):94–6.

478. Zairi I, Mzoughi K, Jnifene Z, Fennira S, Ben Moussa F, Kammoun S, et al. A giant right atrial myxoma with pulmonary arterial hypertension. Pan Afr Med J. 2015;21:96–96.

479. Zeppellini R, Gheno G, Cucchini F. Misleading Echo‐Doppler Findings in Prolapsing Right Atrial Myxoma: Frame‐By‐Frame Analysis and Hemodynamic Observations. Echocardiography. 1995;12(1):85–91.

480. Zheng G sheng, Song G ying, Hu L xia, Lin Z qiong. DIAGNOSIS OF ATRIAL MYXOMA: A CLINICAL ANALYSIS OF 10 CASES. Chinese Medical Journal. 1982;95(03):209–14.
